# Calnexin promotes glioblastoma progression by inducing protective mitophagy through the MEK/ERK/BNIP3 pathway

**DOI:** 10.7150/thno.105591

**Published:** 2025-01-27

**Authors:** Xuchen Liu, Jiangli Zhao, Qingyuan Sun, Zhiwei Xue, Ziyi Tang, Wenyu Liu, Junzhi Liu, Baojian Miao, Nan Su, Yanya He, Yuehua Zhu, Bin Huang, Ning Yang, Chao Li, Jiwei Wang, Xinyu Wang

**Affiliations:** 1 Department of Neurosurgery, Qilu Hospital, Cheeloo College of Medicine and Institute of Brain and Brain-Inspired Science, Shandong University, 250012 Jinan, China.; 2 Jinan Microecological Biomedicine Shandong Laboratory and Shandong Key Laboratory of Brain Function Remodeling, 250117 Jinan, China.; 3 School of Medicine, Cheeloo College of Medicine, Shandong University, 250012 Jinan, China.

**Keywords:** calnexin, glioblastoma, mitophagy, nimodipine, temozolomide

## Abstract

**Rationale:** Glioblastoma multiforme (GBM), one of the most malignant tumors of the central nervous system, has a poor prognosis, mainly because of its high recurrence caused by the rapid development of drug resistance to postoperative chemotherapy. Although macroautophagy/autophagy is believed to be a fundamental factor in tumor survival during chemotherapy, there is still a lack of autophagy biomarkers for predicting patient prognosis and chemotherapeutic efficacy in clinical practice.

**Methods:** We combined transcriptomic and single-cell sequencing data to identify differentially expressed autophagy-related genes in gliomas. Overexpression of calnexin (CANX), a key gene related to protein folding, and its secretion in the endoplasmic reticulum (ER) was identified, suggesting poor prognosis in GBM patients. The autophagy flow related to CANX was detected by transmission electron microscopy (TEM), Western blotting, and immunofluorescence. Flow cytometry, cell proliferation, activity assays, and the GBM intracranial xenograft mouse model were employed to validate CANX's role in GBM progression.

**Results:** CANX knockdown inhibited proliferation and autophagosome formation in GBM cells. On the other hand, CANX overexpression increased mitogen-activated protein kinase (MAPK) activity, leading to the accumulation of BNIP3 (CL2/adenovirus E1B 19 kDa interacting protein 3, a critical factor regulating mitophagy) and protective mitophagy. Notably, when combined with temozolomide (TMZ), CANX knockdown extended the lifespan of GBM-bearing mice. Additionally, our studies revealed that the classic calcium inhibitor nimodipine (ND) decreased CANX expression and thus enhanced the sensitivity to TMZ.

**Conclusions:** Our findings indicate that CANX functions as an oncogene in GBM. We also characterize the CANX/MEK/ERK/BNIP3 mitophagy pathway, provide new insights into the molecular mechanism of GBM drug resistance, and identify a therapeutic target.

## Introduction

Glioblastoma multiforme (GBM) is a high-grade malignant glioma (grade IV) originating from astrocytes. GBM is highly invasive and has a poor prognosis, with a median survival time of approximately 15 months 1. The primary treatment remains surgical resection. Adjuvant radiotherapy and chemotherapy are given after surgery to prevent recurrence 2, 3. Despite these interventions, GBM frequently recurs within a year after surgery, primarily due to the lack of effective adjuvant therapies capable of eradicating residual tumor cells 4.

Autophagy, a critical evolutionarily conserved intracellular process, is involved in the recovery of cellular components to provide essential elements for cell survival 5. Cell starvation and stress can promote autophagy, resulting in the degradation of organelles and proteins in a lysosome-dependent manner 6. Autophagy has been demonstrated to act as a double-edged sword in tumor cells. On the one hand, autophagy is a survival mechanism and prevents tumor initiation in healthy tissues, but it also helps tumor cells avoid damage by drugs and radiotherapy. Mitophagy, a selective autophagy, involves the specific degradation of damaged or dysfunctional mitochondria and maintains mitochondrial dynamics and cellular homeostasis. Numerous autophagy-related (ATG) proteins and multiple signaling pathways regulate cellular autophagy. To date, many genes have been found to act as key regulators of autophagy and thus play roles in different physiological and pathological processes. Our previous studies identified BCL2L13 as an important autophagy-related gene that can induce mitochondrial fission and promote protective mitochondrial phagocytosis 7.

Due to the inability of common compounds to easily pass through the blood‒brain barrier (BBB), temozolomide (TMZ), a DNA alkylating agent, is still used for conventional chemotherapy after GBM surgery 8, 9. TMZ, which causes single- and double-strand DNA breaks and cell cycle arrest, ultimately resulting in tumor cell death, is the only chemotherapeutic agent demonstrated to improve overall survival in GBM patients. Although many treatment strategies, such as immunotherapy, have been proposed to prevent the development of TMZ resistance, overcoming this problem is still difficult. Numerous factors contribute to the development of TMZ resistance. Recent studies suggest that the induction of protective autophagy in GBM cells is a critical mechanism of TMZ resistance 10. Previous studies have confirmed that gliomas can evade the killing effect of TMZ by activating intracellular pathways to increase autophagy levels or by remodeling the tumor microenvironment through extracellular vesicles 11, 12. Moreover, drugs that target and inhibit protective autophagy can effectively improve the sensitivity of GBM cells to chemotherapy. However, the relationship between autophagy-related genes and GBM and the underlying molecular mechanisms remains unknown.

By integrating glioma data from The Cancer Genome Atlas (TCGA) and Genotype-Tissue Expression (GTEx) databases, we identified differentially expressed autophagy-related genes in GBM. Through predictive analyses, we found that calnexin (CANX) is one of the genes most closely associated with glioma progression. CANX is a conserved molecule universally expressed in all cells containing ER membranes 13. It is distributed in different subdomains of the ER membrane and can form contacts with other organelles and cellular structures, such as ribosomes, smooth ER, mitochondria, and nuclear membranes, to regulate intracellular functions 14. As a molecular chaperone, CANX can clear misfolded proteins through the calmodulin/calreticulin pathway 15. In recent years, other specific functions of CANX have been revealed. CANX can form a stable complex with FAM134B to induce endoplasmic reticulum autophagy (ER-phagy) 16, offset the damage caused by endoplasmic reticulum stress (ER-stress), and maintain cellular functions under stress conditions. CANX has also been identified as a marker for various tumors, such as lung cancer 17. These findings suggest that CANX is an initial autophagy-related gene that can serve as a cancer biomarker. However, the function of CANX in glioma has not been fully characterized.

In this study, we present our findings demonstrating an association between CANX expression levels and the malignant grade of glioma, along with prognosis. Research confirmed that CANX can induce protective autophagy, which promotes GBM cell proliferation. Moreover, we confirmed that CANX can activate the MEK/ERK/BNIP3 pathway to achieve resistance to TMZ chemotherapy through mitophagy, indicating that CANX is a potential therapeutic target for GBM. Considering the molecular functions of CANX, we successfully counteracted CANX-mediated protective mitophagy via the classic calcium antagonist nimodipine (ND), suggesting the potential to inhibit autophagy-induced chemoresistance in GBM.

## Methods

### Data acquisition and normalization

The bulk RNA expression information was obtained from TCGA (https://www.cancer.gov/ccg/research/genome-sequencing/tcga) and CGGA (http://www.cgga.org.cn/). Considering the issue of small sample size of normal tissue in TCGA, we utilized the RNA expression data of normal brain tissue from GTEx database (https://www.gtexportal.org/home/), and merged with TCGA data before batch correction. The single cell sequencing data of IDH-wildtype glioblastoma was download from GEO database (https://www.ncbi.nlm.nih.gov/geo/), the detailed accession number is GSE131928. Public immunohistochemistry slides were from the Human Protein Atlas (HPA, https://www.proteinatlas.org/) database. To remove the batch effect, we utilized the function “normalizeBetweenArrays” in “limma” package to merge TCGA and GTEx cohorts.

### Obtaining of autophagy-related genes (ARGs) and differential expression analysis

The ARGs were obtained from Human Autophagy Database (Http://www.autophagy.lu), a total of 222 ARGs were extracted. The differential expression analysis was conducted based on “limma” package in R (version 3.56.2)1. The differential analysis used the Wilcoxon test, and corrected using False Discovery Rate (FDR).

### Survival and enrichment analysis

The univariate and multivariate Cox analyses were conducted using R package “survival” (version 3.5-7). The survival curves and forest plot were plotted by “survminer” (version 0.4.9). The enrichment analysis was conducted using R package “clusterProfiler” (version 4.8.3)3, a P < 0.05 was considered statistically significant.

### Processing of single cell sequencing data

This process was based on the R package “Seurat” (version 4.3.0.1)2. First, we created a Seurat object using the “CreateSeuratObjec” function. After removing cells with nFeatureRNA < 50 or percentage.mt > 10, we used the “CellCycleScoring” function to calculate the cell phase score and mitigate the impact of the cell cycle on the analysis. The data was then normalized using “NormalizeData”, and “FindVariableFeatures” was utilized to identify the top 2000 most variable genes for subsequent PCA dimensionality reduction. We used the first 30 principal components (PCs) to calculate the proximity distance, setting the resolution at 1. Finally, each cluster was annotated using commonly used markers to determine the cell type (T/NK cells: CD3D, CD3E, NKG7; Oligodendrocytes: MOG, MAG, MBP; Monocytes/Macrophages: CD14, CD68; Malignant cells: SOX9, SOX2). The calculation of autophagy and MAPK signaling scores for each malignant cell was conducted using “AddModuleScore” function in “Seurat”.

### Drug sensitivity analysis

The drug sensitivity analysis was conducted using data from the Genomics of Drug Sensitivity in Cancer 2 (GDSC2) database (https://www.cancerrxgene.org/). The relationship between the CANX expression and temozolomide sensitivity was investigated using the “oncoPredict” R package.

### Cell lines and cultures

NHA, GBM#P3, GBM#BG5 and luciferase-stable GBM#P3 were kindly provided by Prof. Rolf Bjerkvig (University of Bergen). Human glioma cell lines T98G, U118MG, A172, and LN229 were purchased from the National collection of Authenticated Cell Cultures (Shanghai, China; Catalog number: SCSP-5274, TCHu216, TCHu171, TCHu244). T98G, U118MG, A172, LN229 and NHA cells were cultured in Dulbecco modified Eagle medium (DMEM; Thermo Fisher Scientific, SH30022.01B) supplemented with 10% fetal bovine serum (FBS, GE Healthcare Life Sciences, 10082147). GBM#P3, GBM#BG5, GBM#BG7 cells were cultured in Neurobasal Medium (Thermo Fisher Scientific, Gibco™, 21103049) containing B27 supplement (20 μL/mL), FGF (20 ng/mL) and EGF (20 ng/mL) for cell culture and passage.

### Small molecule drugs applied in this study

3-MA (Selleck, S2767), Baf (SigmaAldrich, B1793), C16-PAF (CAS 74389-68-7), Nimodipine (Selleck, S1747), PD98059 (Selleck S1177) and TMZ (Selleck, S1237 were dissolved in DMSO (Sigma-Aldrich, D2650) and diluted to working concentrations in a culture medium.

### Preparation of small interfering RNA, overexpression plasmid and stable knockdown virus

Human siRNAs for CANX, BNIP3, ATG5 and BECN1 were designed to silence the corresponding gene and overexpressed plasmid was constructed to overexpress the expression of CANX (GenePhama, Shanghai), the relevant siRNA sequence can be found in Table S3. Lentiviral vectors targeting human shRNA were designed to construct stable knockdown and overexpression of CANX in glioma cell lines (shCANX:5'-CCAAGCCUCUCAUUGUUCATT-3' GenePharma Shanghai Genechem, Shanghai).

### Cell counting kit (CCK)-8 assay

Cell counting kit-8 (CCK-8; Yeasen, 40203ES) was assessed to check the cell viability of glioma cells. Cells (2.0 × 10^4^ cells/well) were seeded into 96-well plates and incubated at 37 °C. After the necessary processing, cells were incubated for an additional 4 h at 37 °C with 10μL of CCK-8 in 100μL DMEM without serum. The absorbance at 450 nm was measured using a microplate reader (Bio-Rad, model 680; Hercules, CA, USA).

### EdU incorporation assay

EdU incorporation Assay (Yeasen, 40275ES76) was applied for cell proliferation check. Culture cells to the logarithmic growth phase, then treat with 10 µM EDU for 2 h. Fix cells with 4% PFA for 15 min, followed by permeabilization with 0.5% Triton X-100 for 20 min. Perform the Click-iT reaction according to the kit instructions, incubating in the dark for 30 min. Optionally, stain nuclei with Hoechst 33342 for 5min. Wash cells with 5%FBS between each step. Labeled cells were examined under fluorescence microscopy and quantified.

### Transmission electron microscope

Cells were fixed with electron microscope fixative (2.5% glutaraldehyde, servicebio). Then fixed with 1% OsO4 in 0.1 M cacodylate buffer for 2 h. Samples were then stained with 1% Millipore-filtered uranyl acetate, dehydrated in increasing ethanol concentrations, and infiltrated and embedded in epoxy resin (ZXBR, Spon 812). Electron photomicrographs were taken from GBM cells' ultrastructures using a transmission electron microscope (JEM-1200EX II, JEOL; Tokyo, Japan).

### Immunofluorescence

After treatment, cells were fixed with 4% paraformaldehyde in PBS (Beyotime Biotechnology, ST476), permeabilized with 0.5% Triton X-100 (Beyotime Biotechnology, ST795) in PBS, and incubated with mouse anti-LC3B antibody (1:200; Cell Signaling Technology, 83506) and rabbit anti-CANX antibody (1:400; abcam, ab22595) in 5% bovine serum albumin (BOSTER, AR0004) in PBS overnight. Primary antibody was detected with Goat Anti-Mouse IgG H&L (Alexa Fluor® 488) (Abcam, ab150177) and Goat Anti-Rabbit IgG H&L (Alexa Fluor® 594) (Abcam, ab150088). Cells were incubated in the dark with DAPI to stain nuclei. Slides were examined under fluorescence microscopy, and images were acquired using a CCD (charge-coupled device) digital camera (Olympus, DP71; Waltham, MA, USA).

### Immunohistochemistry

Paraffin-embedded samples were sectioned (4 µm) and mounted on microscopic slides. Heat-induced epitope retrieval was performed in EDTA antigen repair solution (Solarbio) in a microwave. Sections were incubated with the primary antibody at 4 °C overnight (LC3B, 1:200, Cell Signaling Technology, 12741S; Ki67, 1:200, Cell Signaling Technology, 9027; CANX, 1:1000, Abcam, ab22595; cleaved caspase 3,1:400, Cell Signaling Technology, 9661T), rinsed with PBS, and incubated with horseradish peroxidase-linked goat anti-rabbit secondary antibody (ZSGB-BIO, PV-9000). Visualization was achieved using diaminobenzidine (ZSGB-BIO, ZLI-9033) as the substrate, and slides were counterstained with Mayer hematoxylin (Beyotime Biotechnology, C0107).

### Quantitative real-time PCR

Total RNA was extracted from cells using a kit from Yishan Bio, China, and cDNA was synthesized using the reverse transcription qPCR RT Kit from TOYOBO, Japan. RNA from glioma and normal brain samples was extracted using TRIzol. qRT-PCR was performed using qPCR SYBR Green Master Mix (Yeason, 11201ES) on the 480II Real-Time PCR Detection System (Roche). β-actin mRNA expression was used as a standard, and the results represent at least three independent experiments.

### Western blotting

Protein samples were lysed with RIPA (P0013B, Beyotime) and quantified, then mixed with SDS sample buffer and boiled for 5 min. Equal amounts of protein were loaded onto an SDS-PAGE gel and electrophoresed. Proteins were transferred to a nitrocellulose or PVDF membrane for 1-2 h at 4 °C. Membranes were blocked in 5% non-fat dry milk in TBST for 1 h, followed by incubation with primary antibodies overnight at 4 °C. Membranes were incubated with HRP-conjugated secondary antibodies for 1 h at room temperature. Post-secondary incubation, membranes were washed with TBST and treated with ECL detection reagent, then visualized using an imaging system. The following antibodies were used to incubate membranes: ERK1/2 and MEK1/2 antibody (CST, MAPK Family Antibody Sampler Kit 9926), Phospho-p44/42 MAPK antibody (CST, 4370S), Phospho-MEK1/2 (CST,2338S), CANX antibody (Abcam, ab22595), BNIP3 antibody (Proteintech, 68091-1-Ig), LC3B antibody (CST, 43566S), SQSTM1/p62 antibody (CST, 39749), GAPDH antibody (CST, 5174S), ATG7 antibody (CST, 2631T), BECN1 antibody (Servicebio, GB112053-100), PARP antibody (CST, 9542), cleaved-PARP (CST, 5625), Caspase 3 (CST, 9662) and cleaved Caspase (CST, 39661).

### Mito-Tracker and GFP-LC3B transfection and evaluation

Mito-Tracker (Beyotime, C1049B) with red fluorescence (excitation 550 nm) was used for labeling mitochondria. GFP-LC3B (pBABEpuro, 22405) expression vectors were obtained from Addgene and purified using the EndoFree Plasmid Maxi Kit (QIAGEN, 12362). Mitophagy rate was determined by calculating the average number of GFP-LC3B puncta and RFP-mito merged puncta per cell across more than three highly recognizable fields.

### Detection of *in vitro* mitophagy using mito-Keima fluorescent reporter

The mito-Kemia (mt-Kemia, Hanbio, China) is a mitochondrially localized pH-sensitive ratiometric fluorescent protein probe that allows differential imaging of cytoplasmic and lysosomal mitochondrial localization. The green fluorescence (excitation 440 nm) was used to value the normal mitochondria in basic or neutral conditions and the red fluorescence was used to visualize mitochondria undergoing mitophagy (acidic conditions). Perform confocal imaging on GBM#P3 and T98G cells labeled with mt Keima after corresponding treatment. Quantify the data using ImageJ software by dividing the total number of red pixels by the total number of green pixels.

### Dual-luciferase reporter assay

Predict the NRF1 binding CANX promoter sequence through the Jaspar website, construct the CANX sequence on the pGL3 Basic Vector reporter gene vector, and design the NRFI binding sequence mutation vector (Mut-1/2). Transfect GBM#P3 cells (80% fusion degree) with the luciferase reporter plasmid, internal reference reporter plasmid, and NRF1 overexpression plasmid (h-NRF1)/empty vector (pcDNA3.1). After 48 h, lyse the cells and measure the luciferase activities (firefly and Renilla luciferase activities) using a microplate reader according to measure and normalized the luciferase activity with the requirements of the kit (Biotime, RG029M).

### Fluo-4 Calcium Assay

The Fluo-4 Calcium Assay Kit (S1061M, Beyotime) is used for detecting intracellular calcium ion concentrations. Remove the culture medium and wash the cells with PBS. Add a 100 μl of Fluo-4 staining solution to each 24-well plate well. Incubate at 37 °C in the dark for 30 min. Incubation time can be adjusted between 10-60 min. After incubation, used PBS to wash the cells 2 times and observed the staining under a fluorescence microscope or detected with a fluorescence microplate reader (Fluo-4 AM exhibits green fluorescence, Ex/Em = 490/525 nm).

### GBM Intracranial xenograft mice model

Athymic mice (male; 4 weeks old; ~22 g) were provided by Shanghai SLAC Laboratory Animal Co., Ltd (Shanghai, China). The mice were anesthetized with chloral hydrate and secured on a stereotactic frame. A longitudinal incision was made in the scalp, and a 1 mm-diameter hole was drilled 2.5 mm lateral to the bregma. Luciferase-stable GBM#P3 glioma cells (2 × 10^5^) in 20 μL of serum-free DMEM were implanted 2.5 mm into the right striatum using a Hamilton syringe. Mice were monitored by bioluminescence imaging every week. Briefly, mice were injected with 100 mg luciferin (Caliper, 122796), simultaneously anesthetized with isoflurane, and subsequently imaged with a cooled charge-coupled device camera (IVIS-200, Xenogen; Alameda, CA, USA). Bioluminescence values of tumors were quantified using the Living Image 2.5 software package (Xenogen). Mice were euthanized after 28 days and perfused with 4% paraformaldehyde in PBS. Brains were coronally sectioned for immuno-histochemistry assays.

### Statistical analysis

Three independent experiments were performed, and results were expressed as the mean ± the standard deviation (SD). Data were compared using paired Student t tests for two-group comparison and one-way analysis of variance (ANOVA) for multi-group comparisons in GraphPad Prism 8 software (San Diego, CA, USA). Kaplan-Meier survival curves were generated and compared using the log-rank test. P values determined from different comparisons < 0.05 were considered statistically significant and are indicated as follows: *P < 0.05; ** P < 0.01; ***P < 0.001.

## Results

### CANX was identified as an autophagy-related oncogene in GBM

**Figure 1:**
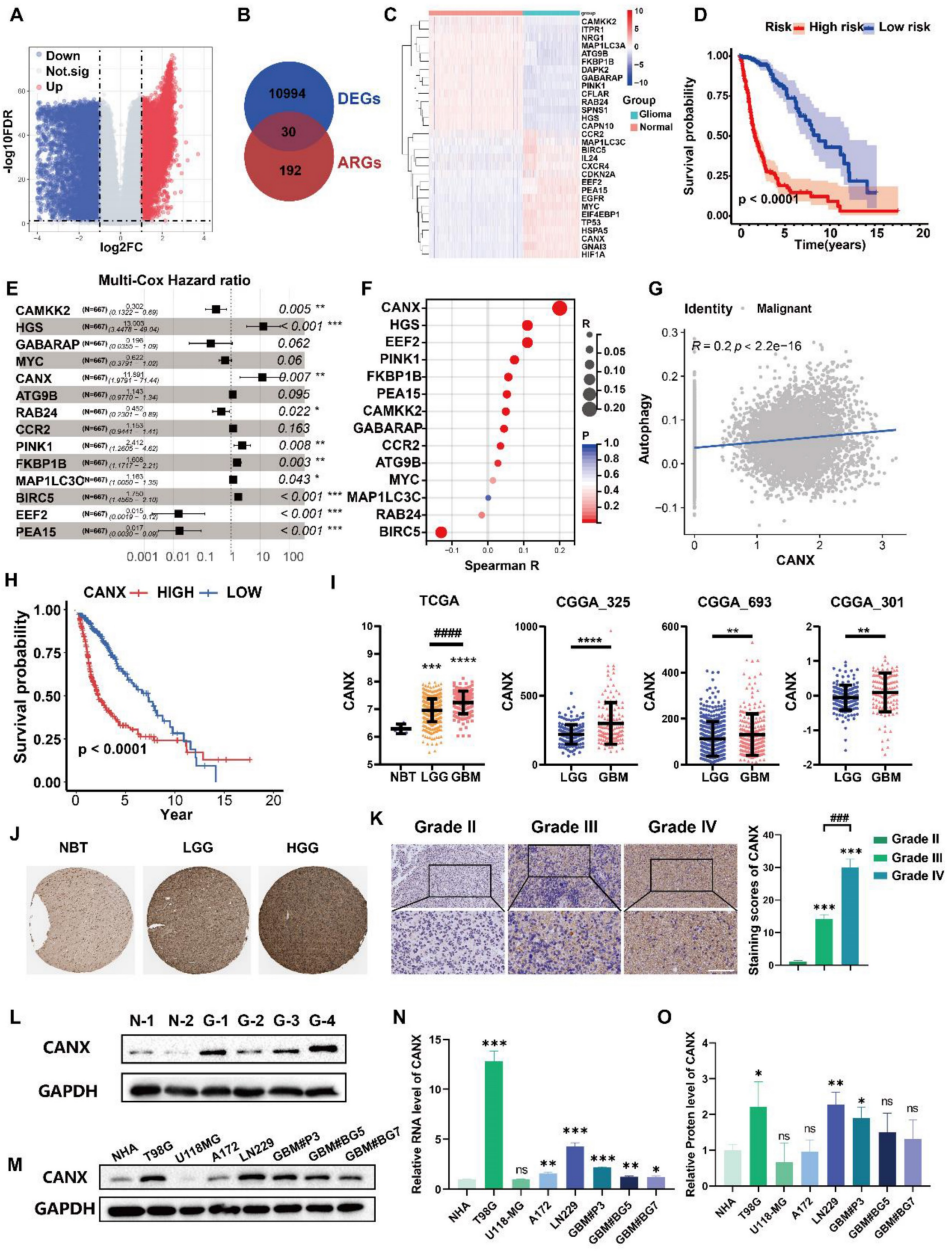
** CANX is an Autophagy-Related Gene that is Differentially Expressed in Gliomas and is Associated with Glioma Prognosis. (A)** A volcano plot showing the DEGs in gliomas identified by data from the TCGA and GTEx databases. **(B)** A Venn diagram showing the intersection of DEGs with ARGs. **(C)** Clustering heatmap of DE-ARGs. **(D)** Prognostic model for glioma patients encompassing DE-ARGs on the basis of TCGA data. **(E)** Multivariate Cox regression analysis revealed the hazard ratios for each independent prognostic indicator gene. **(F)** Autophagy scores were calculated from a single-cell database. **(G)** Correlation analysis between CANX expression and autophagy. **(H)** Kaplan‒Meier survival curves were plotted on the basis of TCGA data for survival analysis of the high and low CANX expression groups. **(I)** Analysis of CANX expression in normal brain tissue (NBT) and tissues from patients with gliomas of different grades according to data from the TCGA and CGGA databases. **(J)** Representative images and statistical analysis of IHC staining results for CANX according to data from the HPA database. **(K)** Images of IHC staining for CANX in gliomas of different grades (scale bar: 100 μm). **(L)** Western blot showing the difference in CANX expression between NBT and glioma tissues. **(M)** Immunoblotting results showing CANX expression in normal human astrocytes and various glioma cells. **(N)** qPCR results showing the RNA expression levels of CANX in NHAs and different glioma cell lines. **(O)** Statistical analysis of normalized CANX protein levels on the basis of the Western blot results. The data are shown as the means ± SDs and are representative of three independent experiments. *P < 0.05; **P < 0.01, ***P < 0.001, ****P < 0.0001 between the two indicated treatments.

Using information from the TCGA and GTEx databases and the “limma” package, we identified differentially expressed genes (DEGs), including 5,636 downregulated and 5,632 upregulated in tissues from glioma patients compared with normal brain (Figure 1A). The Venn diagram showed the intersection of these DEGs with autophagy-related genes (ARGs), yielding 30 differentially expressed autophagy-related genes (DE-ARGs) in glioma (Figure 1B). The heatmap of the RNA expression of the DE-ARGs between normal and glioma tissues identified 16 upregulated and 14 downregulated genes (Figure 1C). We subsequently performed survival and univariate Cox regression analyses (Table S1). Using data from the TCGA database, we constructed a prognostic model for glioma patients, demonstrating that individuals classified as high risk had significantly poorer outcomes than those classified as low risk (Figure 1D). Multivariate Cox regression analysis was used to determine the hazard ratios for each independent prognostic indicator, and the results are illustrated in Figure 1E.

We performed single-cell sequencing analysis to identify key genes affecting autophagy in malignant cells and found that malignant cells expressed high levels of SOX2 and SOX9 (Figure S1A-C). We calculated autophagy scores for each cell line and found that these scores strongly correlated with CANX expression (Figure 1F-G, Figure S1D). We further evaluated the potential role of CANX as an oncogene across various cancers. Differential expression analysis revealed that CANX was highly expressed in most tumor types across datasets, including LGG, GBM, and complete glioma (LGGGBM) (Figure S2A). Survival analysis revealed that CANX was associated with a greater risk of most cancers, with the LGGGBM group exhibiting the most significant p-value (Figure S2B). Based on data from the Chinese Glioma Genome Atlas (CGGA), we analyzed the IDH mutation status and 1p/19q codeletion in GBM patients. Our analysis revealed that CANX expression was relatively higher in IDH wild-type patients and that high CANX expression was frequently associated with 1p/19q codeletion (Figure S2C-D). These findings indicated that CANX may significantly promote cancer progression and increase the glioma risk.

Glioma cells were divided into high- and low-CANX expression groups (Figure S1E- F). Survival analysis of data from the TCGA database confirmed that CANX was a risk factor for glioma prognosis (Figure 1H). Its expression was elevated in glioma tissues and positively correlated with tumor grade across data from the TCGA, Chinese Glioma Genome Atlas (CGGA) (Figure 1I), and Human Protein Atlas (HPA) databases (Figure 1J). Immunohistochemical staining of glioma specimens from Qilu Hospital further validated these findings (Figure 1K). Western blot analysis revealed that CANX expression was generally elevated in glioma tissues (Figure 1L). Additionally, we assessed CANX expression in human normal astrocytes (NHAs), glioma cell lines (T98G, U118MG, A172, and LN229 cell lines), and primary GBM cells (GBM#P3, GBM#BG5, and GBM#BG7 cells) using Western blotting and qPCR (Figure 1M-O). CANX was highly expressed in most glioma cells except U118-MG cells. Notably, CANX expression was markedly higher in T98G cells, resistant to TMZ chemotherapy, than in other glioma cells.

### CANX knockdown inhibited proliferation and promoted apoptosis in GBM cells

**Figure 2:**
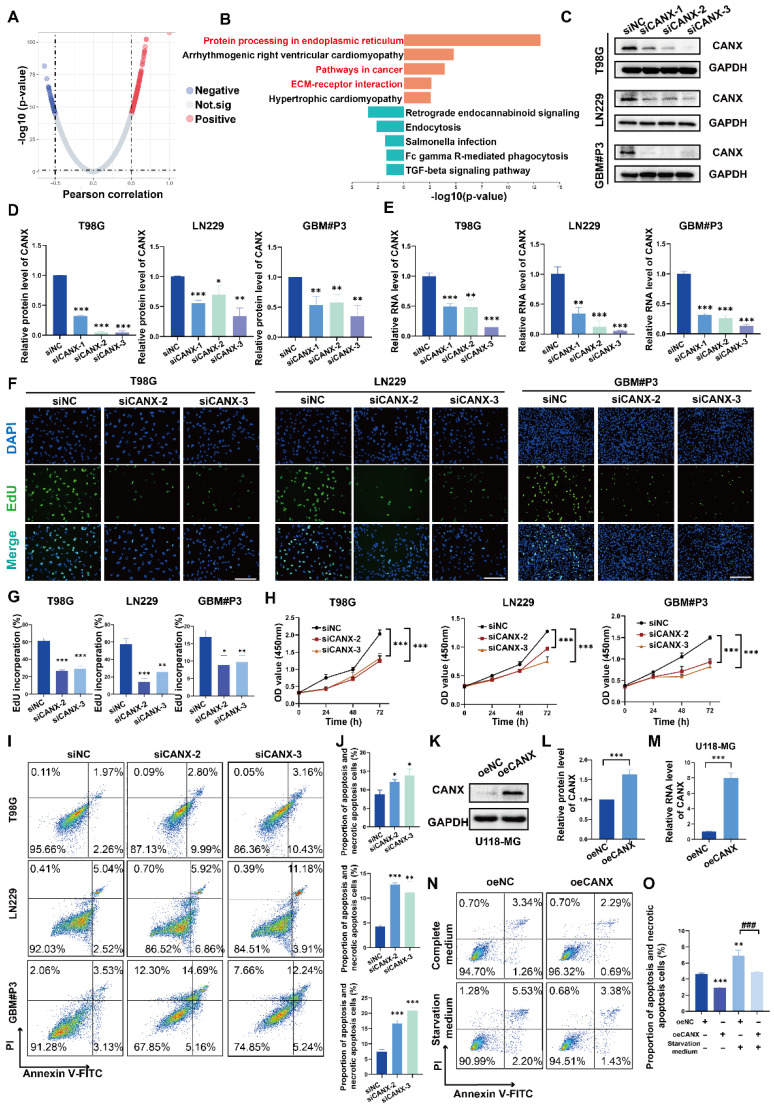
** Knockdown of CANX Inhibits GBM Cell Migration and Promotes GBM Cell Apoptosis. (A)** Volcano plot showed the DEGs identified based on the TCGA data. **(B)** KEGG enrichment analysis of the DEGs. **(C)** Analysis of the knockdown efficiency of three siRNAs targeting CANX in T98G, LN229, and GBM#P3 cells via Western blotting. **(D)** Statistic results of the knockdown efficiency of three siRNAs via Western blotting. **(E)** Analysis of the knockdown efficiency of three siRNAs targeting CANX in T98G, LN229, and GBM#P3 cells via qRT‒PCR. **(F)** EdU staining assay of T98G, LN229, and GBM#P3 cells treated with siCANX and siNC (scale bar: 100 μm). **(G)** Statistic results of the EdU staining assay of T98G, LN229, and GBM#P3 cells treated with siCANX and siNC. **(H)** A CCK-8 assay was used to analyze cell viability of T98G, LN229, and GBM#P3 cells with CANX knockdown. **(I, J)** Flow cytometry analysis of propidium iodide (PI) and Annexin V-FITC staining in T98G, LN229, and GBM#P3 cells transfected with siNC, siCANX-2, and siCANX-3 for the analysis of apoptosis and the statistic results of the proportion of apoptosis cells and necrotic apoptosis. **(K, L)** Validation of the efficiency of the CANX overexpression lentivirus via Western blotting in U118-MG cells and the statistic results of the relative protein level of CANX in U118-MG cells. **(M)** Validation of the efficiency of the CANX overexpression lentivirus via qRT‒PCR. **(N, O)** Analysis of apoptosis in CANX-overexpressing cells under normal and starvation conditions in U118-MG cells and the statistic results of the proportion of apoptosis cells and necrotic apoptosis cells. The data are shown as the means ± SDs and are representative of three independent experiments. *P < 0.05; **P < 0.01, ***P < 0.001 between the two indicated treatments.

Positively and negatively correlated genes with CANX expression were identified via a volcano map (Figure 2A). KEGG enrichment analysis suggested that CANX was highly enriched in multiple pathways related to tumor growth, such as ER protein synthesis, cancer pathways, and ECM-receptor interaction mechanisms (Figure 2B). Following small interfering RNA (siRNA) treatment, CANX levels in T98G, LN229, and GBM#P3 cells were verified via quantitative real-time PCR (qRT‒PCR) and Western blotting (Figure 2C-E). The results of the EdU-DNA synthesis assay demonstrated that lower expression levels of CANX led to the inhibition of DNA replication in T98G, LN229, and GBM#P3 cells (Figure 2F-G). The results of the CCK-8 assay suggested that the viability of GBM cells significantly decreased after CANX knockdown (Figure 2H). Flow cytometry apoptosis assays confirmed that the apoptosis and necrotic apoptosis rate of T98G, LN229, and GBM#P3 cells with CANX knockdown was significantly increased (Figure 2I-J). A plasmid was used to generate CANX-overexpressing U118-MG cells; subsequently, CANX overexpression was verified via qRT‒PCR and Western blotting (Figure 2K-M). The apoptosis rates of U118-MG cells in the normal complete medium and starvation medium were then assessed via flow cytometry apoptosis assays after treatment with the overexpression plasmid.

Western blot analysis further validated the effect of common apoptotic markers, Caspase 3, cleaved Caspase 3, PARP, and cleaved PARP on apoptosis. The results showed that after CANX knockdown, T98G, LN229, and GBM#P3 cells exhibited varying degrees of upregulation in apoptotic markers (increased cleaved Caspase 3/Caspase 3 and cleaved PARP/PARP ratios). Conversely, CANX overexpression decreased the levels of these apoptotic markers (decreased cleaved Caspase 3/Caspase 3 and cleaved PARP/PARP ratios) (Figure S3A). The results indicated that CANX overexpression protected GBM cells from apoptosis and necrotic apoptosis, with the protective effect being more pronounced under starvation conditions (Figure 2N-O).

### CANX promoted protective autophagy in GBM cells

**Figure 3:**
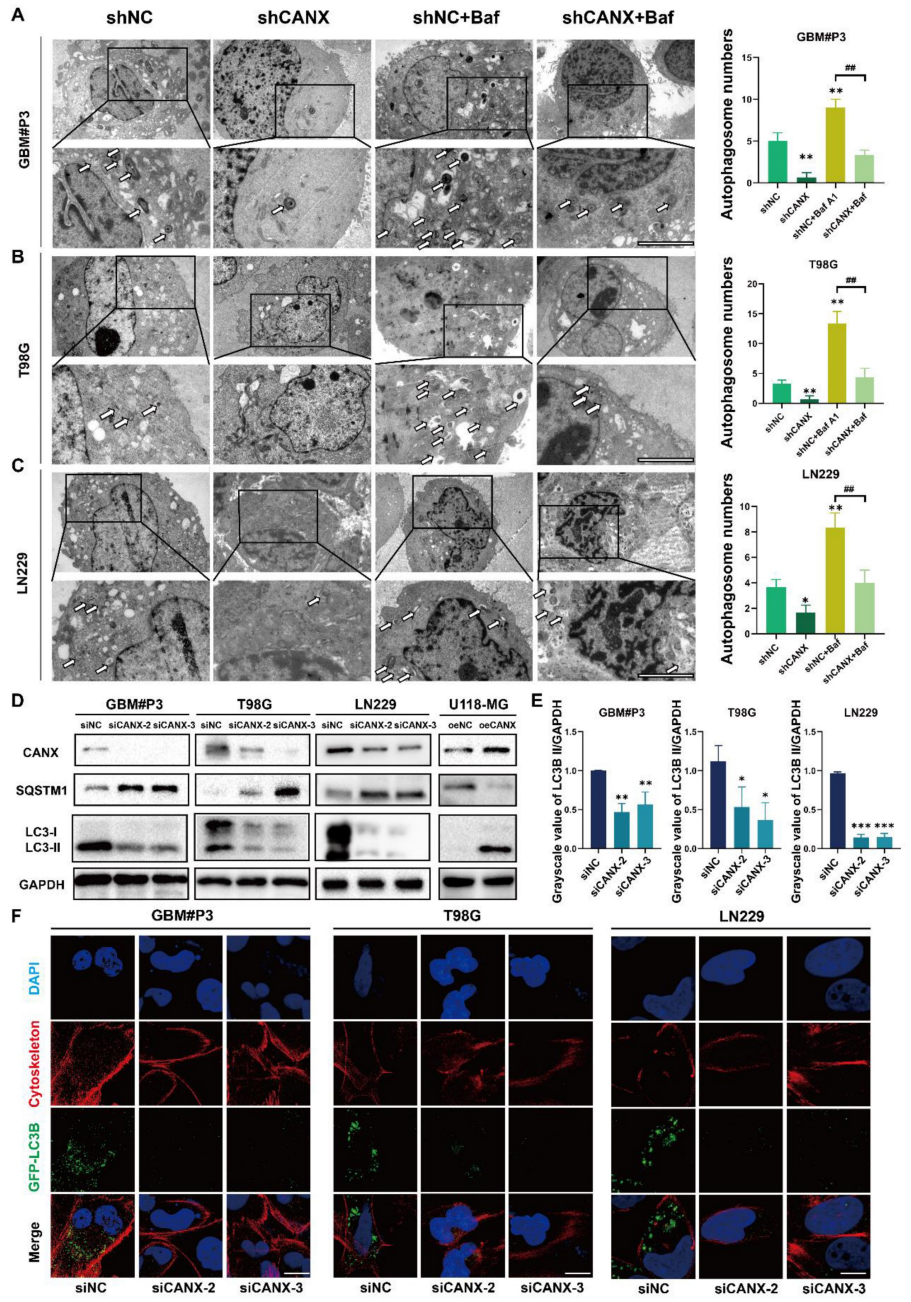
** Knockdown of CANX Inhibits Autophagosome Formation. (A-C)** TEM images showing the changes in the number of autophagosomes in the GBM#P3, T98G and LN229 cell lines following CANX knockdown under normal conditions and after bafilomycin A1 (Baf) treatment (scale bar: 3 μm). The arrows indicate autophagosomes. **(D)** Western blot analysis confirmed the changes in the expression of the autophagy markers LC3B-II and SQSTM1 after CANX knockdown. **(E)** The relative changes in LC3B levels were quantified via Western blot analysis. **(F)** Immunofluorescence staining and confocal microscopy revealed alterations in LC3B expression following CANX knockdown (scale bar: 50 μm). The data are shown as the means ± SDs and are representative of three independent experiments. *P < 0.05; **P < 0.01, ***P < 0.001 between the two indicated treatments.

To further understand the relationship between CANX and autophagy in GBM cells, transmission electron microscopy (TEM) was performed to detect autophagosomes. The results confirmed that the number of autophagic vesicles significantly decreased in T98G, LN229, and GBM#P3 cells with CANX knockdown, and treatment with bafilomycin A1 (Baf), an autophagy inhibitor, further promoted the blockade of autophagosome formation (Figure 3A-C). Western blot analysis suggested that after CANX knockdown, the expression of the autophagy marker MAP1LC3B (LC3B)-II was downregulated. In contrast, the mitophagy marker SQSTM1 expression was upregulated, suggesting that mitophagy was inhibited. The MAP1LC3B-I expression in U118-MG cells was significantly increased by CANX overexpression, whereas SQSTM1 expression decreased (Figure 3D).

Immunofluorescence staining of MAP1LC3B and the cytoskeletal marker phalloidin combined with confocal microscopy were performed to assess the formation of autophagosomes in T98G, GBM#P3, and LN229 cells after knocking down CANX. The statistical results of the gray value ratio between LC3B-II and GAPDH are shown in Figure 3E. LC3 puncta decreased after CANX expression was downregulated (Figure 3F and Figure S3 B-D). Moreover, in U118-MG cells overexpressing CANX, the number of LC3 puncta increased (Figure S3E-F).

**Figure 4:**
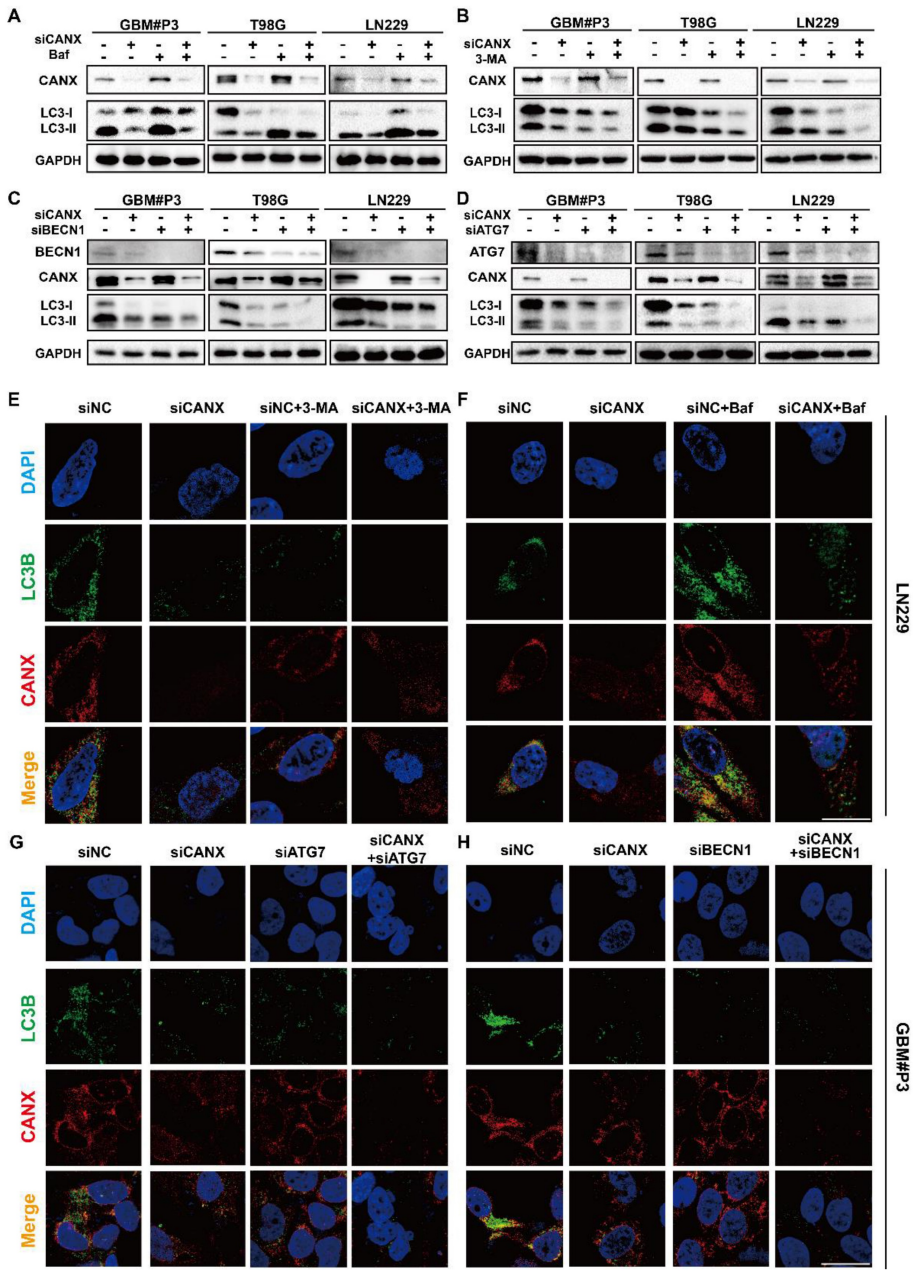
** Validation of the Role of CANX in Regulating Autophagic Flux in GBM. (A, B)** Western blot analysis demonstrated the effect of CANX knockdown on LC3B levels in the presence of the autophagy inhibitors Baf and 3-MA in GBM#P3, T98G an LN229 cells. **(C, D)** The Western blot results validated the impact of CANX knockdown on MAPLC3B-II levels when BECN1 and ATG7 in GBM#P3, T98G an LN229 cells with siCANX and siNC. **(E, F)** Immunofluorescence assays of the LN229 cell line revealed the effect of CANX knockdown on autophagosome formation after treatment with 3-MA and Baf (scale bar: 25 μm). **(G, H)** Confocal microscopy analysis of immunofluorescence in the GBM#P3 cell line was used to assess the impact of CANX knockdown on autophagic flux following the knockdown of ATG7 and BECN1 (scale bar: 25 μm).

To further evaluate the role of CANX in regulating autophagy in GBM, we examined autophagic flux. We treated T98G, LN229, and GBM#P3 cells with the late-stage autophagy inhibitor Baf (100 nM) and the early-stage autophagy inhibitor 3-MA (10 mM) for 48 h. Western blot analysis revealed that LC3B-II accumulation after Baf treatment decreased when CANX expression was reduced (Figure 4A). In contrast, the decrease in LC3B-II accumulation after 3-MA treatment was more pronounced upon CANX knockdown (Figure 4B). Besides knocking down CANX expression, we also transfected T98G, LN229, and GBM#P3 cells with siRNAs targeting ATG7 and BECN1 to inhibit their expression. The results indicated that silencing BECN1 and ATG7 in the three cell lines decreased the LC3B-II protein level. Further knocking down CANX resulted in a more pronounced decrease in the LC3B-II level (Figure 4C-D). Subsequently, immunofluorescence staining of CANX (red fluorescence) and LC3B (green fluorescence) was performed. The changes in the number of LC3B puncta in LN229 and GBM#P3 cells observed under a microscope after treatment with 3-MA and Baf, as well as the knockdown of BECN1 and ATG7, were consistent with the Western blot results (Figure 4E-H and Figure S4A-B).

**Figure 5:**
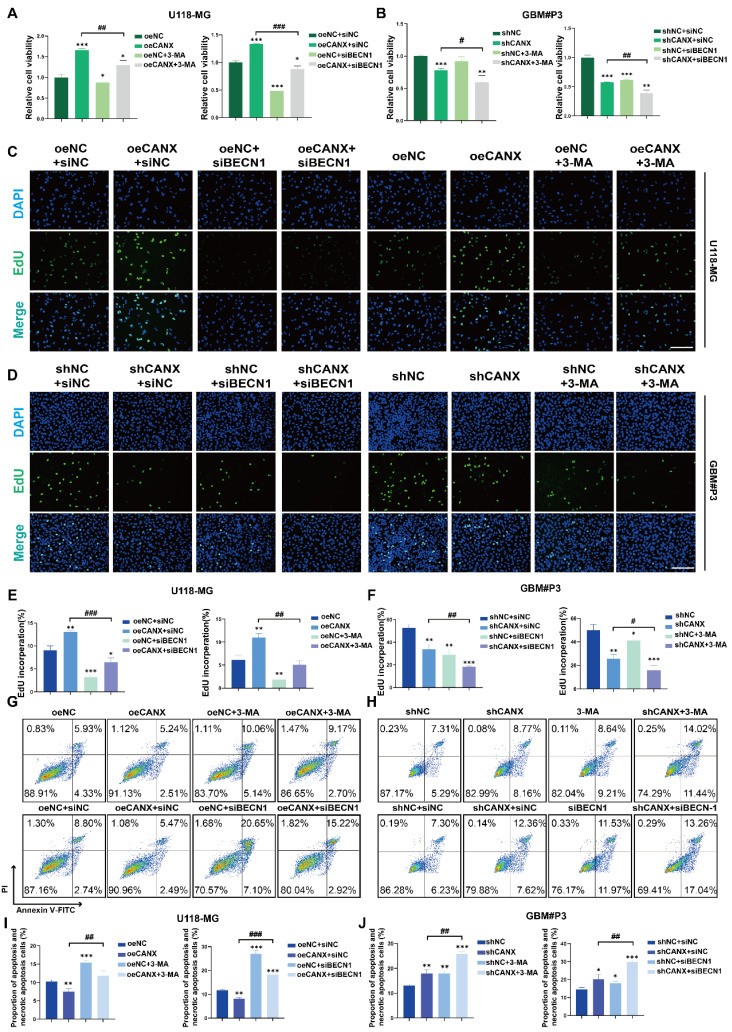
** CANX Overexpression Promotes Protective Autophagy in GBM Cells *In Vitro*. (A, B)** Assessment of the viability of NC-overexpressing/CANX-overexpressing U118-MG cells and shNC/shCANX-treated GBM#P3 cells after incubation with 3-MA or siBECN1 via the CCK-8 assay. **(C, D)** Representative images of the EdU staining assay of NC-overexpressing/CANX-overexpressing U118-MG cells and shNC-treated/shCANX-treated GBM#P3 cells after 3-MA or siBECN1 incubation (scale bar: 100 μm). **(E, F)** Statistical analysis of the EdU staining assay of NC-overexpressing/CANX-overexpressing U118-MG cells and shNC-treated/shCANX-treated GBM#P3 cells after 3-MA or siBECN1 incubation. **(G, H)** Flow cytometry analysis of Annexin V-FITC and PI staining in NC-overexpressing/CANX-overexpressing U118-MG cells and shNC-treated/shCANX-treated GBM#P3 cells after 3-MA or siBECN1 incubation for the analysis of apoptosis. **(I, J)** Statistic results of the proportion of apoptosis U118-MG and GBM#P3 cells and necrotic apoptosis after 3-MA or siBECN1 incubation. *P < 0.05, **P < 0.01, and ***P < 0.001 between the two indicated treatments.

Cell viability was assessed after 3-MA treatment and BECN1 knockdown using the CCK-8 assay to determine whether CANX-induced autophagy protects tumor cells. When 3-MA was added, and BECN1 was knocked down in CANX-overexpressing U118-MG cells, cell viability was decreased to normal levels (Figure 5A). We found that 3-MA treatment or BECN-1 knockdown led to further decreases in the viability of GBM#P3 cells after CANX was knocked down (Figure 5B). EdU assays also revealed that the inhibition of autophagy led to the offset of increase in cell proliferation caused by CANX overexpression in U118-MG cells (Figure 5C, E) and further increases in the inhibition of proliferation in shCANX-treated GBM#P3 cells (Figure 5D, F). Also, flow cytometry apoptosis assays revealed that 3-MA or BECN1 knockdown increase the apoptosis rate of oeCANX-treated U118-MG cells (Figure 5G, I). Besides, the increase in the apoptosis rate of ShCANX-GBM#P3 cells was further promoted (Figure 5H, J). These results indicated that CANX induces protective autophagy in GBM cells.

### CANX-mediated mitophagy promoted TMZ resistance in GBM cells

**Figure 6:**
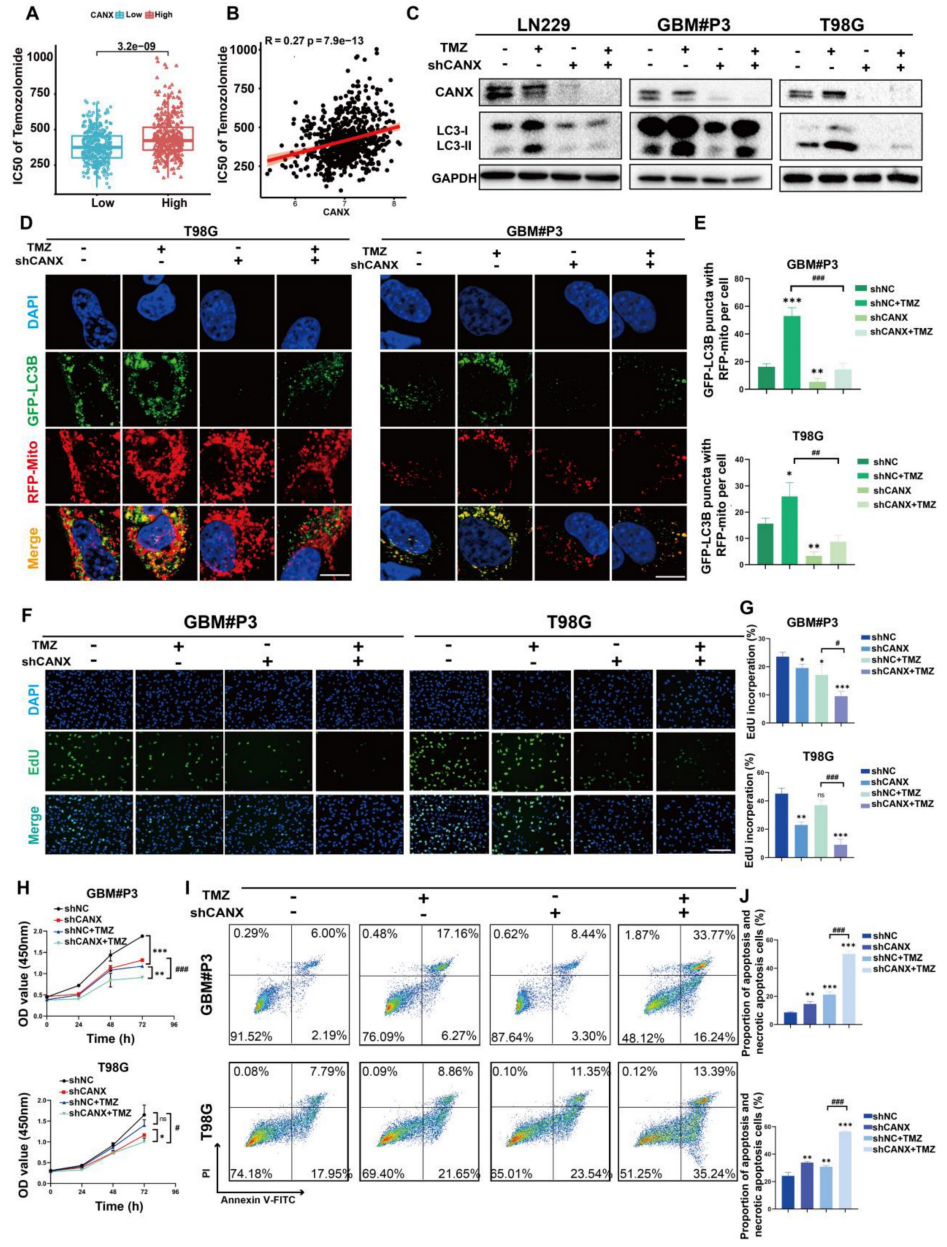
** Inhibition of CANX Enhances Sensitivity to TMZ Chemotherapy. (A)** Comparison of the IC50 values of TMZ between cells with high and low CANX expression on the basis of RNA expression data from the TCGA database. **(B)** Correlation between CANX expression levels and the IC50 values of TMZ in glioma cells. **(C)** Western blot analysis showing the inhibition of TMZ-induced autophagy following CANX knockdown. **(D)** Confocal microscopy images of GFP-LC3B and RFP-mito illustrating mitophagy under TMZ treatment and CANX knockdown in T98G and GBM#P3 cells (scale bar, 40 µm). **(E)** Statistic results of GFP-LC3 puncta with RFP-Mito per cell under TMZ treatment and CANX knockdown in T98G and GBM#P3 cells. **(F, G)** EdU assay and statistic results showing the effect of reduced CANX expression on TMZ efficacy (scale bar: 100 μm). **(H)** CCK-8 assay showing the effect of CANX inhibition on sensitivity to TMZ. **(I, J)** Apoptosis assays demonstrating the impact of reduced CANX expression on TMZ-induced apoptosis with statistic results in GBM#P3 and T98G cells. The data are shown as the means ± SDs and are representative of three independent experiments. *P < 0.05; **P < 0.01, ***P < 0.001 between the two indicated treatments.

To determine whether the inhibition of autophagy by CANX knockdown affects the sensitivity of GBM cells to TMZ, we calculated the IC50 of TMZ for each patient on the basis of RNA expression information from the TCGA database. The results revealed that glioma patients with increased expression of CANX had increased IC50 values (Figure 6A-B), which suggests that patients with increased expression of CANX require increased doses of TMZ to achieve successful chemotherapy intervention. The Western blot results revealed that TMZ (100 μmol/L) significantly upregulated LC3B in the GBM cell lines and that CANX knockdown inhibited this upregulation of LC3B (Figure 6C). Previous experiments have confirmed that TMZ can effectively induce mitophagy in glioma cells 18. We examined the expression levels of several common mitophagy markers, such as Parkin, PINK1, NDP52, and Optineurin, to investigate whether CANX knockdown inhibits mitophagy in GBM cells and validate the relationship between CANX and mitophagy. The results showed that the expression of all four markers was downregulated in the siCANX group, suggesting that CANX expression may upregulate mitophagy (Figure S7F). We also investigated the effect of CANX knockdown on mt-Keima reporter gene expression. In GBM#P3 and T98G cells treated with siCANX, the fluorescence intensity under 550 nm excitation was significantly reduced, while that under 440 nm excitation was increased, indicating impaired mitophagy (Figure S7C-E). These results suggest that mitophagy levels significantly decreased following CANX knockdown in GBM cells.

To further confirm whether CANX affects mitophagy, we used markers for mitochondria and GFP-LC3B to assess the colocalization of autophagosomes with mitochondria. We found the upregulation of mitophagy by TMZ could be inhibited by knocking down CANX (Figure 6D-E). To determine whether knocking down CANX further promotes the cytotoxic effects of TMZ in GBM cells, EdU and CCK-8 assays were used to assess proliferation and cell viability (Figure 6F-H Figure S5D, F). The results showed that the combination of CANX knockdown with TMZ further enhanced the cytotoxic effects of TMZ. Additionally, flow cytometry of apoptosis assays revealed that TMZ significantly increased apoptosis rates after CANX was knocked down in T98G and GBM#P3 cells (Figure 6I-J). These findings that knocking down CANX can sensitize GBM cells to TMZ were verified in the LN229 cell line (Figure S5A-C, E).

### CANX knockdown enhanced TMZ chemosensitivity in an orthotopic mouse model

**Figure 7:**
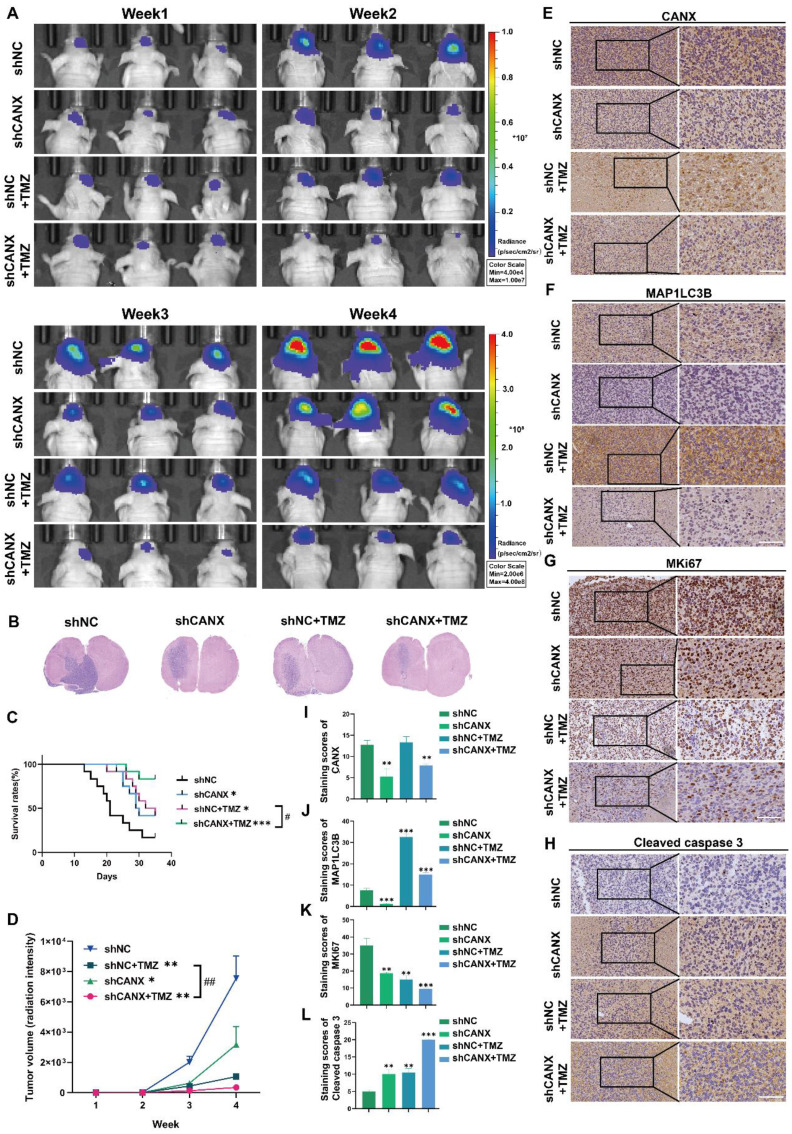
** CANX Knockdown Enhances the Efficacy of TMZ in an Orthotopic GBM Mouse Model. (A)** GBM#P3 cells expressing luciferase were orthotopically implanted into immunosuppressed nude mice, and tumor growth was monitored via the IVIS-200 imaging system. Bioluminescent signals were measured on days 7, 14, 21, and 28 post-implantation. **(B)** HE staining showing the extent of tumor invasion within the brain. **(C)** Overall survival was determined via Kaplan‒Meier survival curves, with statistical significance assessed via the log-rank test. **(D)** Bioluminescence values for assessing tumor growth. **(E-L)** Immunohistochemical staining images and statistical analysis of MKi67, LC3B, CANX, and cleaved caspase 3 expression in tumors from each group (scale bar: 100 μm) **(E-L)**. The data are shown as the means ± SDs and are representative of three independent experiments. *P < 0.05; **P < 0.01, ***P < 0.001 between the two indicated treatments.

We constructed an orthotopic tumor model in nude mice to further elucidate the role of CANX in GBM development. We generated luciferase-stable GBM#P3 cells transduced with shNC (n = 24) or shCANX (n = 24) and treated half of the experimental animals in each group (n = 12) with TMZ (20 mg/kg/once per day) via gavage. Tumor growth was monitored by bioluminescence imaging (Figure 7A). The results revealed that tumor progression was significantly slower in the shCANX group than in the shNC group. Hematoxylin and eosin (HE) staining demonstrated that the loss of CANX reduced GBM cell proliferation and invasion (Figure 7B). Kaplan‒Meier survival analysis revealed a statistically significant difference in prognosis between the shCANX and shNC groups, with the combination of shCANX and TMZ resulting in a better prognosis than TMZ or shCANX alone (Figure 7C). Moreover, radiation intensity analysis revealed that the combination of TMZ and shCANX achieved better efficacy than TMZ treatment alone (Figure 7D).

The histochemical staining for CANX confirmed that the shCANX group had low CANX expression (Figure 7E, I). Additionally, the expression level of MAP1LC3B was highest in the TMZ group and significantly decreased after CANX knockdown (Figure 7F, J). The histochemical staining also revealed that the expression level of MKI67, which is indicative of proliferative capacity, was lowest in the shCANX+TMZ group (Figure 7G, K). In contrast, the expression level of cleaved Caspase 3, indicative of apoptosis, was highest in this group (Figure 7H, L). These results demonstrated that the knockdown of CANX significantly enhances the ability of TMZ to suppress GBM cell growth and prolong survival, suggesting that the inhibition of CANX expression can effectively augment the antitumor effect of TMZ in glioma.

### CANX drives BNIP3-mediated protective mitophagy

**Figure 8:**
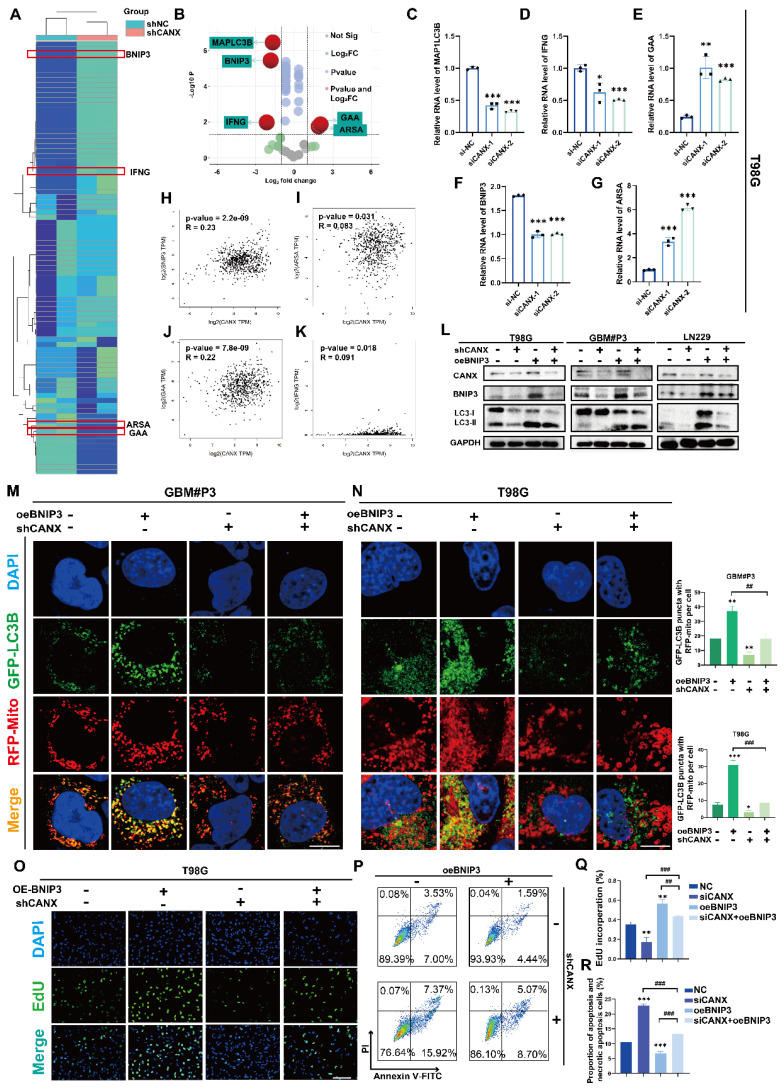
** CANX Regulates GBM Progression by Modulating BNIP3-Mediated Mitophagy. (A, B)** A cluster heatmap and volcano plot of the PCR data showing significantly DE-ARGs between the shCANX and shNC groups. **(C-G)** PCR validation of the expression levels of these DEGs (IFNG, GAA, BNIP3, ARSA and MAP1LC3B) in the T98G cell line after the knockdown of CANX. **(H-K)** Correlation analysis of single-cell data was performed to determine the correlations between CANX expression and GAA, BNIP3, IFNG and ARSA expression. **(L)** Western blot showing the expression levels of LC3B, BNIP3 and CANX in shCANX-treated and shNC-treated T98G, GBM#P3 and LN229 cells overexpressing BNIP3. **(M, N)** Confocal microscopy images of GFP-LC3B and RFP-mito were used to reveal changes in mitophagy following CANX knockdown and BNIP3 overexpression in T98G and GBM#P3 cells (scale bar: 20 μm) . **(O)** EdU assay showing the effect of BNIP3 overexpression on shCANX-treated and shNC-treated T98G cells' proliferation (scale bar: 100 μm). **(P)** Flow cytometry analysis showing the impact of BNIP3 overexpression on apoptosis in T98G cells. **(Q)** The statistical analysis of the EdU assay with the treatment of BNIP3 overexpression on shCANX-treated and shNC-treated T98G cells. **(R)** Statistic results of the proportion of apoptosis and necrotic apoptosis T98G cells with oeBNIP3 and shCANX treatment. The data are shown as the means ± SDs and are representative of three independent experiments. *P < 0.05; **P < 0.01, ***P < 0.001 between the two indicated treatments.

In an attempt to further understand the potential mechanisms by which CANX regulates autophagy, we identified the autophagy-related genes (Figure S6A) in GBM#P3 cells with stable CANX knockdown and compared them with normal control cells using an RT‒PCR array. A clustering heatmap and a volcano plot were used to identify key autophagy-related genes with significant differences in expression between the two groups (log FC > 1 or < -1 and P < 0.05) (Figure 8A-B). Among the statistically significant DEGs identified, MAP1LC3B, IFNG, GAA, BNIP3 and ARSA were differentially expressed in T98 cells, consistent with the sequencing results (Figure 8C-G). Based on TCGA data, we performed correlation analysis and detected a significant positive correlation between CANX and BNIP3 expression; however, there was a negative correlation between GAA and ARSA expression and a weaker correlation between CANX and IFNG expression (Figure 8H-K). As a classic marker of mitophagy 19, BNIP3 was significantly downregulated upon CANX knockdown, suggesting that it may be a downstream target of CANX. Western blot analysis was performed to confirm whether CANX expression influences the accumulation of BNIP3. The results revealed that the upregulation of LC3B following BNIP3 overexpression could be inhibited by knocking down CANX in T98G, GBM#P3, and LN229 cells (Figure 8L).

We used fluorescently labeled markers for mitochondria (RFP-mito) and autophagosomes (GFP-LC3B) to ascertain whether CANX affects mitophagy mediated by BNIP3 and assess the colocalization of autophagosomes with mitochondria in both GBM cell lines. Confocal microscopy revealed the accumulation of MAP1LC3B puncta and increased colocalization of RFP-mito and GFP-LC3B following BNIP3 overexpression, which was inhibited upon CANX knockdown (Figure 8M-N, Figure S7A-B). Previous studies have suggested that BNIP3 can mediate protective autophagy, enabling GBM cells to evade TMZ-induced cell death 18. An EdU-DNA synthesis assay demonstrated that BNIP3 overexpression induced protective autophagy in glioma cells, whereas reducing CANX expression in BNIP3-overexpressing cells adversely affected their proliferative ability (Figure 8O, Q; Figure S6B-E). Flow cytometry apoptosis assays revealed that BNIP3 overexpression reduced CANX-induced apoptosis (Figure 8P, R; Figure S6F-G). In summary, our preliminary findings suggest that CANX expression regulates the activation of BNIP3-mediated protective mitophagy in GBM cells.

### CANX regulated mitophagy via activating the MEK/ERK/BNIP3 pathway

**Figure 9:**
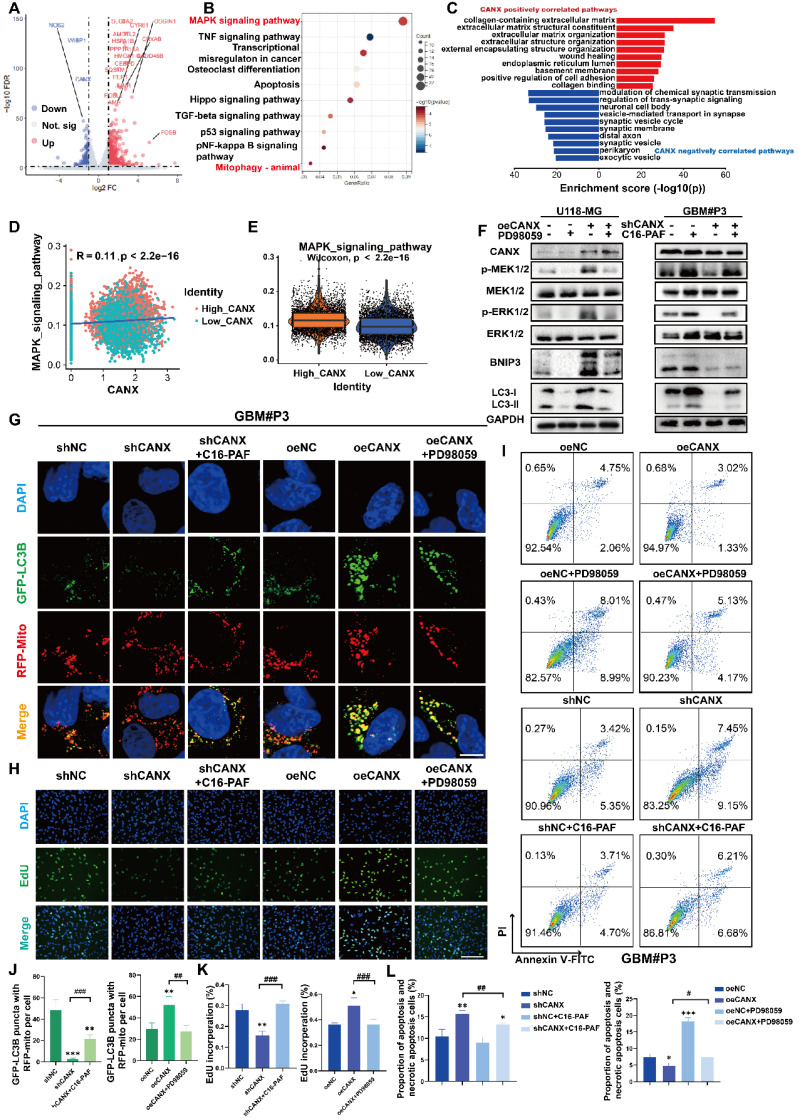
** The MAPK Pathway Plays a Role in CANX-Mediated Regulation of Protective Mitophagy. (A)** Volcano plot showing DEGs identified by whole-transcriptome sequencing of GBM#P3 cells in the shCANX group and shNC group. **(B)** KEGG enrichment analysis of these DEGs. **(C)** GO enrichment analysis of CANX-related DEGs according to data from the TCGA database. **(D)** Correlation analysis between MAPK and CANX expression. **(E)** Differences in MAPK expression between GBM cells with high and low CANX levels. **(F)** Western blot showing the protein levels of MEK, p-MEK, ERK, p-ERK, LC3B, BNIP3, CANX and GAPDH after treatment with PD98059 and C16-PAF in CANX-overexpressing U118-MG cells and shCANX-treated GBM#P3 cells. **(G)** Confocal microscopy images of GFP-LC3B and RFP-mito showing mitophagy changes after MAPK pathway agonist and inhibitor treatment in GBM#P3 cells (scale bar: 10 μm). **(H)** EdU assay demonstrating the impact of CANX and MAPK pathway alterations on GBM#P3 cells' proliferation (scale bar: 100 μm). **(I)** Flow cytometry analysis showing the impact of PD98059 and C16-PAF to assess the effects of changes in CANX expression on U118-MG and GBM#P3 cells' apoptosis level. **(J)** Statistic results of GFP-LC3 puncta with RFP-Mito per cell after PD98059 and C16-PAF treatment in GBM#P3 cells. **(K)** The statistical analysis of the EdU assay with the treatment of PD98059 and C16-PAF in GBM#P3 cells. **(L)** Statistic results of the proportion of apoptosis and necrotic apoptosis GBM#P3 cells with PD98059 and C16-PAF treatment. The data are shown as the means ± SDs and are representative of three independent experiments. *P < 0.05; **P < 0.01, ***P < 0.001 between the two indicated treatments.

We performed sequencing of mRNA isolated from shCANX- and shNC-transfected GBM#P3 cells in an attempt to identify DEGs regulated by CANX in GBM cells. By using the “limma” package, 526 genes were identified whose expression was positively correlated with CANX expression and 234 genes negatively correlated with CANX. The DEGs are displayed in a volcano plot (Figure 9A). Additionally, we constructed a protein-protein interaction (PPI) network of DEGs and identified the top 20 hub genes using the MCC algorithm (Figure S8A-B). We conducted GO enrichment analysis and the results showed that CANX is related to the postive regulation of intracellular biosynthesis and other processes (Figure S8C-E). KEGG enrichment analysis indicated that CANX was most strongly enriched in the MAPK pathway. The results also suggested that CANX was related to apoptosis and mitophagy in animals (Figure 9B).

In conjunction with TCGA data analysis, we applied GO enrichment analysis of the CANX-related DEGs and found that CANX is enriched with the extracellular matrix, the ER lumen and positive regulation of cell adhesion (Figure 9C). These results support a possible association between CANX and the progression of GBM. The findings of previous studies and the above results 20, 21 indicate that the MAPK signaling pathway is an important signaling pathway involved in the CANX-mediated development of GBM. Using single-cell sequencing data, we calculated the MAPK scores of each malignant cell type in glioma brain tissue. The results suggested that the CANX expression level was positively correlated with the MAPK score in glioma cells (Figure 9D-E).

Previous studies suggested a close correlation between the MEK/ERK pathway and the regulation of BNIP3 expression 22. Therefore, we used Western blotting to examine phosphorylated levels of MEK1/2 and ERK1/2, MEK1/2, ERK1/2, LC3B, and BNIP3 in shCANX-GBM#P3 cells after treatment with the MEK agonist C16-PAF and in CANX-overexpressing U118-MG cells following treatment with the MEK inhibitor PD9805. Phosphorylated MEK and ERK levels decreased when CANX was knocked down and increased when CANX was overexpressed. When C16-PAF was applied, BNIP3 and LC3-II levels were increased, and the opposite results were observed when PD98059 was used (Figure 9F). Furthermore, immunofluorescence analysis suggested that after blocking the MEK/ERK axis, the level of mitophagy induced by CANX overexpression decreased. In contrast, the mitophagy level increased in shCANX-treated cells following C16-PAF treatment (Figure 9G, J). We further investigated whether CANX-mediated phosphorylation of MEK/ERK was involved in inhibiting proliferation and promoting apoptosis in GBM cells. EdU staining confirmed that the MAPK pathway is crucial for CANX-mediated regulation of tumor proliferation (Figure 9H, K). PD98059 increased apoptosis in GBM#P3 cells overexpressing CANX, whereas C16-PAF inhibited apoptosis induced by CANX knockdown (Figure 9I, L).

### ND reduced CANX expression and enhanced sensitivity to TMZ

Previous studies have confirmed that TMZ can induce calcium influx via TRPM2 channels. This process helps maintain mitochondrial function and alleviates the damage to the mitochondrial membrane potential caused by hypoxia and TMZ treatment 23. Moreover, CANX maintains cellular calcium homeostasis by regulating the release and reuptake of Ca^2+^, ensuring normal cellular functions. Additionally, since Ca^2+^ is a crucial secondary messenger in the MAPK pathway 24, 25, CANX indirectly regulates the MAPK pathway by binding Ca^2+^ and modulating their concentrations. Theoretically, reducing CANX expression can lead to decreased calcium storage in the ER, which may inhibit the development of TMZ resistance.

**Figure 10:**
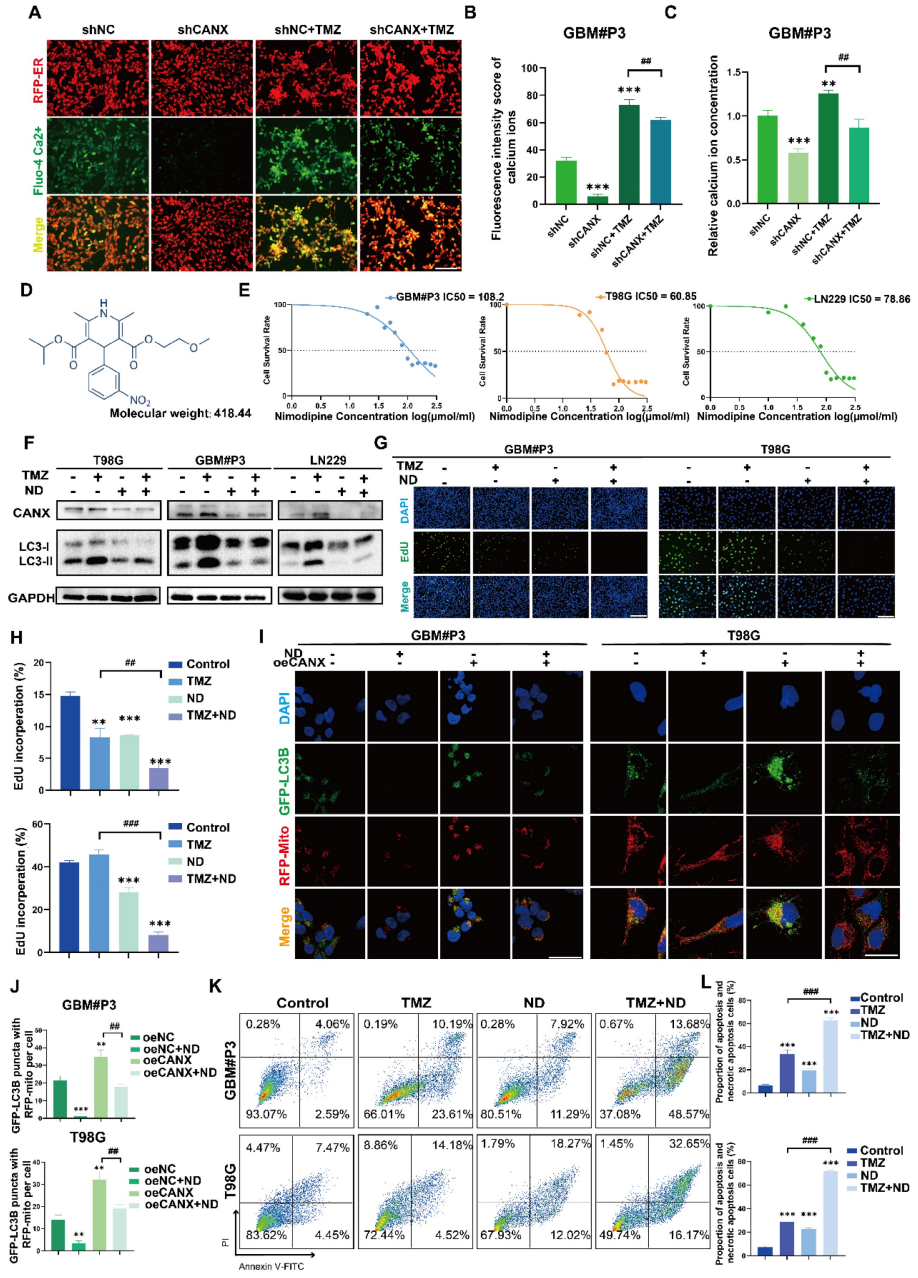
** ND Inhibits CANX-Induced Protective Autophagy by Decreasing Intracellular Calcium Levels. (A)** Fluorescence microscopy showing ER morphology and calcium ion distribution in GBM#P3 cells stained with Fluo-4 Ca²+ and ER-Tracker (scale bar: 80 μm). **(B)** Analysis of the calcium ion fluorescence intensity in GBM#P3 cells (shNC/shCANX group) treated with TMZ. **(C)** Quantitative analysis of the Ca²+ fluorescence intensity in GBM#P3 cells (treated with shNC/shCANX) before and after TMZ treatment via a microplate reader. **(D)** Molecular structure of ND. **(E)** IC50 curves of ND in the GBM#P3, T98G, and LN229 cell lines. **(F)** Western blot analysis showing the protein level of LC3B and CANX in GBM#P3, T98G, and LN229 cells after treatment with TMZ and ND. **(G, H)** EdU assay showing the proliferation of GBM#P3 and T98G cells after treatment with TMZ and ND with statistic results (scale bar: 40 μm). **(I, J)** Representative confocal images showing changes in GFP-labeled LC3 levels in GBM#P3 and T98G cells stained with MitoTracker Red after treatment with ND and TMZ with statistic results of GFP-LC3 puncta with RFP-Mito per cell (scale bar: 100 μm). **(K, L)** Flow cytometry analysis of apoptosis levels in GBM#P3 and T98G cells after treatment with TMZ and ND with statistic results. The data are shown as the means ± SDs and are representative of three independent experiments. *P < 0.05; **P < 0.01, ***P < 0.001 between the two indicated treatments.

We used a red fluorescent probe to label the ER and a green probe to label Ca^2+^. The staining results revealed that in the GBM#P3 and LN229 cell lines, after TMZ treatment, the ER was swollen, and the Ca^2+^ content was increased; however, after CANX knockdown, the Ca^2+^ content in the ER was significantly reduced (Figure 10A, Figure S9B). Additionally, we calculated the fluorescence intensity of Ca^2+^ (Figure 10B, Figure S9C) and performed a quantitative analysis of intracellular Ca^2+^ concentration using an enzyme-linked immunosorbent assay and reached the same conclusion (Figure 10C). These findings suggest that inhibiting the intracellular uptake of Ca^2+^ may have an effect equivalent to that of inhibiting CANX expression. Meanwhile, research found the increasing of intracellular Ca^2+^ concentration can also induce upregulation of CANX protein expression in hippocampal neurons 26. An increase in intracellular Ca^2+^ concentration is one of the triggers of ER stress. In response to ER stress, cells activate the unfolded protein response (UPR). A critical component of UPR is the upregulation of ER chaperone protein expression included CANX 27. Specifically, a series of signaling pathways initiated by UPR sensor proteins ultimately lead to an increase in the transcription and translation of the CANX gene, resulting in an upregulation of CANX protein expression 28. Therefore, calcium channel blockers (CCBs) might inhibit CANX accumulation and interrupt CANX function.

Following the literature, we created a list of common CCBs' brain-to-plasma partition coefficient (Kp), reflecting their ability to cross the BBB (Table S2). We treated T98G and GBM#P3 cells with several CCBs at appropriate concentrations for 48 h, then extracted proteins to assess CANX levels. The results showed that both nimodipine and nifedipine significantly and consistently reduced CANX expression (Figure S9I). Based upon the BBB permeability, we chose ND, the molecular formula of which is shown in Figure 10D, as a specific blocker of CANX for further study. Western blotting confirmed that after 48 h of treatment with 50 μmol/ml ND, the CANX expression level was downregulated in the T98G, LN229, and GBM #P3 cell lines, and the LC3B-II level was also decreased (Figure S9A). A IC50 curve was constructed to determine the concentration at which ND kills GBM cells, and the results suggested that ND could induce cell death at lower concentrations in the T98 cell line than in the other cell lines (Figure 10E). Western blotting confirmed that ND inhibited CANX expression and reduced TMZ-induced LC3B-II accumulation in all three cell lines (Figure 10F). Immunofluorescence further confirmed that ND decreased the accumulation of LC3B puncta and the colocalization of GFP-LC3B and RFP-mito, which suggested that mitophagy in GBM cells was inhibited after ND application (Figure 10I-J; Figure S9D, F). Furthermore, EdU staining revealed that ND exerted a synergistic effect with TMZ, maximizing the ability of TMZ to inhibit tumor cell proliferation while overcoming chemoresistance, in GBM#P3, LN229 and T98G cell lines (Figure 10G-H; Figure S9E, G). Flow cytometry apoptosis assays indicated that ND could induce apoptosis and further increase TMZ cytotoxicity (Figure 10K-L Figure S9H). Moreover, we evaluated the impact of ND in primary GBM cells GBM#BG5 and GBM#BG7. According to the CCK-8 assay, ND similarly increased the chemosensitization of TMZ and suppressed GBM cell proliferation in both of these cell lines (Figure S9J-K).

### NRF1 acts as a transcription factor to promote CANX transcription in GBM cells

Using the hTFtarget database, we searched for transcription factors that were predicted to target the CANX gene and closely associated with its expression, overlapped with those with a score > 20 in the TFDB database, and also associated with CANX in brain tissue in the GTRD database. The analysis predicted NRF1 as the most relevant transcription factor promoter (Figure S10A). The CANX-binding motif of NRF1 is displayed (Figure S10B). We constructed two transcription factor binding sites (TFBSs) of CANX promoter with high scores and corresponding mutations into the pGL3-basic vector (Figure S10C-D). Correlation analysis based on TCGA suggests a significant positive correlation between CANX and NRF1 in GBM patients (Figure S10E). Western blotting and PCR results showed that, compared with the pcDNA3.1 blank group, h-NRF1 enhanced NRF1 expression in GBM#P3 cells (Figure S10F-G). Dual-Luciferase reporter assay indicated significantly elevated luciferase activity in h-CANX (WT)+pcDNA3.1 and h-CANX (WT)+h-NRF1 groups compared with the pGL3-Basic group. The luciferase activity was significantly upregulated in the h-NRF1 group, suggesting that NRF1 can bind to the CANX promoter and participate in CANX transcription. Meanwhile, we also found that the mutation of the two binding sites of NRF1 on the CANX promoter decreased luciferase activities compared with h-CANX (WT). These results suggest that NRF1 binds to the CANX promoter and regulates CANX transcription in both TFBSs (Figure S10H).

### The combination of ND and TMZ induced a stronger chemotherapeutic effect in glioma than TMZ alone

**Figure 11:**
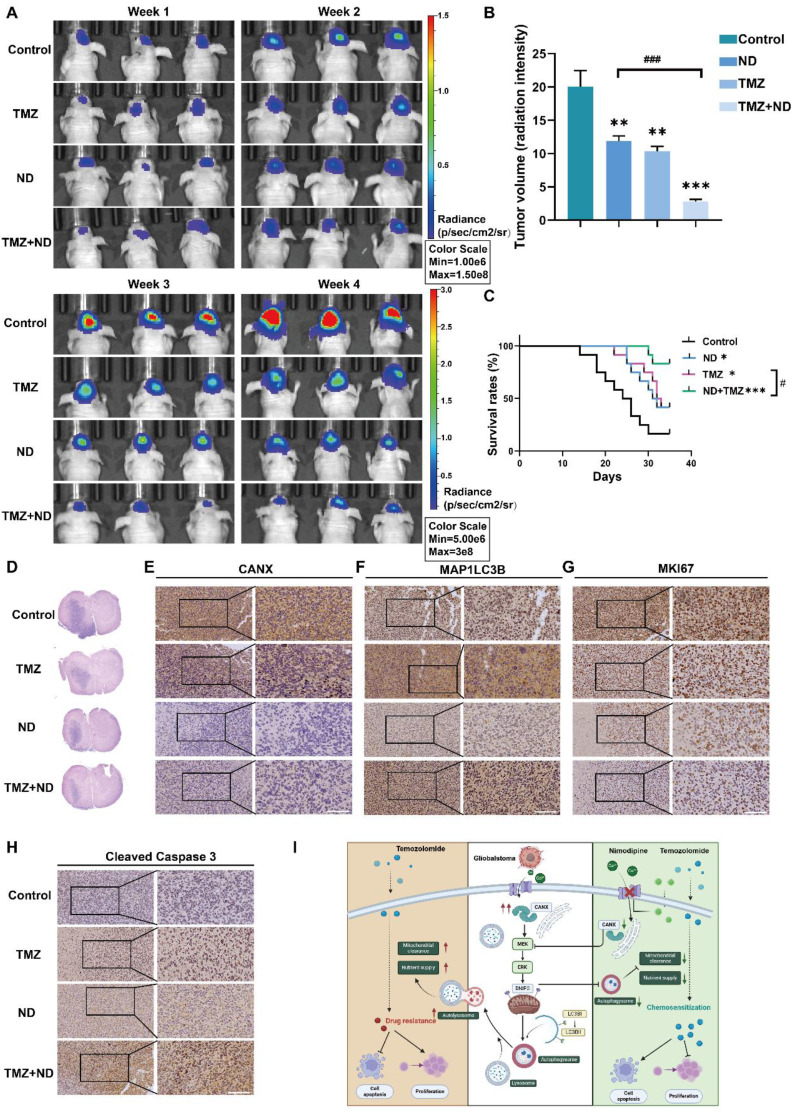
***In Vivo* Experiments Confirm that ND Enhanced the Chemotherapeutic Effect of TMZ. (A)** GBM#P3 cells expressing luciferase were implanted intracranially into immunosuppressed nude mice, followed by treatment with ND, TMZ, or a combination of both. Tumor growth was monitored via the IVIS-200 imaging system, and bioluminescence signals were measured on days 7, 14, 21, and 28 post-implantation. **(B)** Bioluminescence values for assessing tumor growth on day 28. **(C)** Overall survival was determined via Kaplan‒Meier survival curves, and statistical significance was assessed via the log-rank test. **(D)** HE staining showing the extent of tumor invasion in the brain. **(E-H)** Immunohistochemical staining images of MKi67, LC3B, CANX, and cleaved caspase 3 in tumors from each treatment group. **(I)** Study flowchart. The data are shown as the means ± SDs and are representative of three independent experiments. *P < 0.05; **P < 0.01, ***P < 0.001 between the two indicated treatments.

Based on previous studies, we divided 48 tumor-bearing (GBM#P3 cells) nude mice into four groups: 12 mice in the control group received only solvent injections; 12 mice were treated with TMZ (20 mg/kg/once per day) only; 12 mice received ND treatment (according to a previous study, the dosage was 2 mg/kg once per day to achieve a relatively high plasma concentration and increased half-life 29); and 12 mice received a combination of TMZ and ND. Tumor growth was monitored using small animal imaging equipment, and the tumor fluorescence intensity in the fourth week was recorded (Figure 11A-B). The results indicated that ND had a mild inhibitory effect on mouse tumor growth. In contrast, the combination of TMZ and ND resulted in the most significant tumor growth inhibition, suggesting that ND enhances the efficacy of TMZ. The survival curves further corroborated this finding, as longer survival was observed in the combination group than in the groups treated with either agent alone (Figure 11C). After the mice were sacrificed, their brain tissues were sectioned and stained. The HE staining results confirmed the difference in tumor growth among the groups, while staining of CANX, MAP1LC3B, MKI67, and cleaved Caspase 3 confirmed that ND could enhance chemotherapy sensitivity *in vivo* by reducing the expression of CANX (Figure 11D-H, Figure S10 I-L). A schematic diagram is shown in Figure 11I.

## Discussion

CANX, a well-known resident gene of the ER, is considered a key regulator involved in protein assembly 30. Our study revealed that CANX was significantly upregulated in glioma patients. By integrating transcriptome sequencing and single-cell analysis, we observed that CANX expression progressively increased with glioma grade and that higher CANX expression correlated with poorer prognosis. Our results demonstrated that CANX knockdown effectively inhibited the proliferation of GBM cells and induced tumor apoptosis. Based on these findings, CANX is a potential oncogene associated with GBM and may serve as a promising therapeutic target for GBM treatment.

Although the regulatory effect of CANX on autophagy has been reported, the underlying molecular mechanism remained elusive 31. The induction of autophagy by CANX in GBM was demonstrated by TEM, qRT‒PCR, Western blotting and confocal imaging. We found that CANX induced protective autophagy via the application of specific autophagy inhibitors or siRNAs targeting key autophagy-related genes. Moreover, our results are the first to demonstrate that CANX could promote mitophagy mediated by BNIP3. It has been shown that ER stress is associated with the activation of JNK and the upregulation of BNIP3 in cardiac dysfunction 32. As a key regulatory gene for ER-stress, CANX has the potential to regulate the expression and function of BNIP3.

We also investigated the signaling pathway downstream of CANX and identified that CANX regulated the MAPK pathway. The MEK/ERK signaling pathway, a significant component of the MAPK pathway, has been identified as a therapeutic target in tumors. There is also evidence that the MEK/ERK pathway can induce BNIP3 expression 33. Therefore, we hypothesized that the MEK/ERK pathway acts as a link between CANX and BNIP3-mediated mitophagy. We treated GBM cells with specific inhibitors and agonists of the MEK/ERK pathway to validate this hypothesis. The results showed that the knockdown of CANX effectively reduced the phosphorylation of MEK/ERK pathway components. When agonists were applied, BNIP3 accumulated after CANX was downregulated. Confocal microscopy images also confirmed the reactivation of mitophagy after it was inhibited.

Recently, sensitization of GBM to chemotherapeutic agents, particularly TMZ, has been considered crucial for addressing glioma recurrence. Among the various mechanisms influencing TMZ efficacy, the induction of protective autophagy is believed to rescue tumor cells from death by efficiently clearing damaged organelles and providing essential nutrients for growth under stress conditions. Elevated autophagy in tumor cells can lead to a dramatic reduction in the chemotherapeutic effectiveness of TMZ. In this study, we confirmed that CANX knockdown effectively enhanced the efficacy of TMZ chemotherapy using multiple cell viability and proliferation assays. When the expression of CANX was effectively inhibited *in vivo* and *in vitro*, apoptosis of GBM cells significantly increased, and upon exposure to TMZ, GBM cells with lower CANX expression were widely killed. Therefore, we concluded that CANX can sensitize cells to chemotherapy by decreasing TMZ-mediated autophagy.

It's noteworthy that the development of chemoresistance in malignant tumors is closely related to autophagy. Therefore, targeting and inhibiting the activation of protective autophagic pathways is a leading objective in cancer therapy. Recent studies have shown that inhibiting heparanase can suppress tumor growth by inhibiting autophagy, a finding observed across a range of malignancies 34, 35. There was also a study demonstrated that novel HPSE inhibitor can affect the survival of glioma cells by inhibiting autophagy and upregulating apoptosis 36. We believe that designing key autophagy inhibitors to effectively suppress heparanase activity will provide another therapeutic strategy for sensitizing GBM to chemotherapy. The combination of multiple strategies will offer new avenues for developing more promising GBM treatments.

Despite the complex role of autophagy in GBM, multiple studies have provided compelling evidence that the inhibition of autophagy may promote tumor cell apoptosis and necrosis 37, 38. The inhibition of autophagy has been demonstrated to be a promising therapeutic strategy for GBM and to reduce the chemotherapeutic resistance of GBM 39, 40. Although classic autophagy inhibitors such as hydroxychloroquine and CQ have been tested as adjuvants to chemotherapeutics in clinical trials 41, they have shown serious toxic effects, such as skin damage, ototoxicity and nephrotoxicity, after long-term treatment 42, 43. Moreover, some autophagy inhibitors have a limited ability to pass through the BBB and exhibit poor *in vivo* efficacy when applied with other chemotherapeutic agents. Therefore, the development of autophagy inhibitors with fewer clinical side effects and beneficial effects is urgently needed for GBM treatment. There is a bidirectional regulatory relationship between the concentration of Ca^2+^ and the expression of CANX. The absence of CANX can lead to Ca^2+^ homeostasis disorder 44, studies also demonstrated that a decrease in Ca^2+^ levels within the ER can affect the expression levels and activity of ER chaperone proteins included CANX, thereby impacting the protein folding process 45. CANX can interact with calmodulin (CaM), and this interaction is regulated by Ca^2+^ concentration. CaM is an intracellular calcium ion sensor that participates in many cellular signaling pathways. The interaction between CANX and CaM can influence CANX activity and its response to changes in calcium ion concentration 46. Decreased intracellular calcium ion concentration can not only inhibit the UPR-related signaling pathways, achieving negative feedback regulation of CANX, but can also lead to reduced interaction between CaM and CANX, subsequently resulting in a downregulation of the latter's expression. Therefore, selectively inhibiting the uptake of Ca^2+^ by GBM cells and reducing the concentrations of cytoplasmic and ER Ca^2+^ may effectively suppress CANX-mediated protective autophagy. This is the theoretical basis for the search for novel autophagy inhibitors in this study.

Previous studies have shown that CCBs such as verapamil and ND have inhibitory effects on the proliferation of human glioma cells *in vitro* 47. Among these CCBs, ND is widely used in clinical practice due to its excellent ability to cross the BBB and prevent brain edema and vasospasm following craniotomy 48. Moreover, a new study predicted that ND is be efficacious in treating GBM 49. However, whether ND can regulate autophagy and enhance chemotherapy sensitivity is still unclear, and there is a lack of *in vivo* experiments confirming its efficacy against GBM. In our study, ND not only killed multiple GBM cell lines but also effectively inhibited the expression of CANX and Ca^2+^ uptake. One of the most exciting findings of our study is the synergistic effect of ND and TMZ against GBM *in vivo*. We found that ND could effectively inhibit TMZ-mediated protective autophagy and prolong the survival of GBM-bearing mice. Since ND is an FDA-approved drug with limited hypotensive effects and minimal impacts on coronary artery hemodynamics 50, these findings could be easily translated to the clinic.

However, it should be noted that there are currently not enough clinical samples or cohorts to verify the exact effect of ND on the sensitivity of GBM chemotherapy. It is also important to mention that due to the heterogeneity of GBM, different subtypes exhibit varying responses to different chemotherapeutic agents. While this study investigated the sensitivity of GBM cells with TP53 and PTEN mutations to ND and further refined the testing using primary cells, this is insufficient to validate ND's general efficacy against GBM fully. These limitations require further investigation and large-scale, well-designed clinical trials. Notably, our study also identified NRF1 as a transcription factor closely associated with CANX expression, which aligns with prior research. NRF1 has been linked to glioma severity and poor prognosis in glioblastoma. This study further provides a rationale for the association of higher NRF1 expression with adverse survival outcomes and resistance to temozolomide treatment. However, the functions of NRF1 are complex, and the relationship between NRF1 and CANX warrants further investigation.

To summarize, we searched for autophagy-related DEGs in GBM that are capable of predicting prognosis, identified the oncogene CANX as closely related to GBM progression, verified its key role in promoting tumor cell proliferation and its ability to exert antiapoptotic effects by regulating autophagic flow, and elucidated the mechanism of TMZ resistance. Using high-throughput sequencing, we also explored how mitochondrial autophagy is regulated via the ERK/MEK/BNIP3 axis and designed targeted drugs for intervention. Our study identified CANX as an oncogene in GBM. Also, it provided new targets and related drugs for enhancing the sensitivity of gliomas to chemotherapy, possibly aiding the development of adjuvant therapies for gliomas.

## Conclusion

Our findings afford a foundation for the in-depth study of the role of autophagy in GBM and provide novel insights into the mechanism of drug resistance development, laying the foundation for future clinical trials in GBM patients.

## Supplementary Material

Supplementary figures and tables.

## Abbreviations

ATG: autophagy related
ATG7: autophagy related 7 gene
Baf: bafilomycin A1
BBB: blood-brain barrier
BECN1: beclin 1
BNIP3: BCL2/adenovirus E1B 19 kDa interacting protein 3
CANX: calnexin
CGGA: Chinese Glioma Genome Atlas
ER: endoplasmic reticulum
ER-stress: endoplasmic reticulum stress
GBM: glioblastoma
GTEx: Genotype-Tissue Expression
MAP1LC3/LC3: microtubule associated protein 1 light chain 3
MAPK: mitogen-activated protein kinase
NHA: normal human astrocytes
PBS: phosphate-buffered saline
shRNA: short hairpin RNA
siRNA: short interfering RNA
SQSTM1/p62: sequestosome 1
TCGA: The Cancer Genome Atlas
TMZ: temozolomide
ND: nimodipine
3-MA: 3-methyladenine
