# Supplementary material for: Calnexin promotes glioblastoma progression by inducing protective mitophagy through the MEK/ERK/BNIP3 pathway
Source: Theranostics. 2025 Jan 27;15(6):2624–48. doi: 10.7150/thno.105591 (PMC11840740; doi:10.7150/thno.105591)
Supplement: Supplementary file 1 — Supplementary figures and tables. [file thnov15p2624s1.zip › Supplementary Material/Supplementary figures.docx]

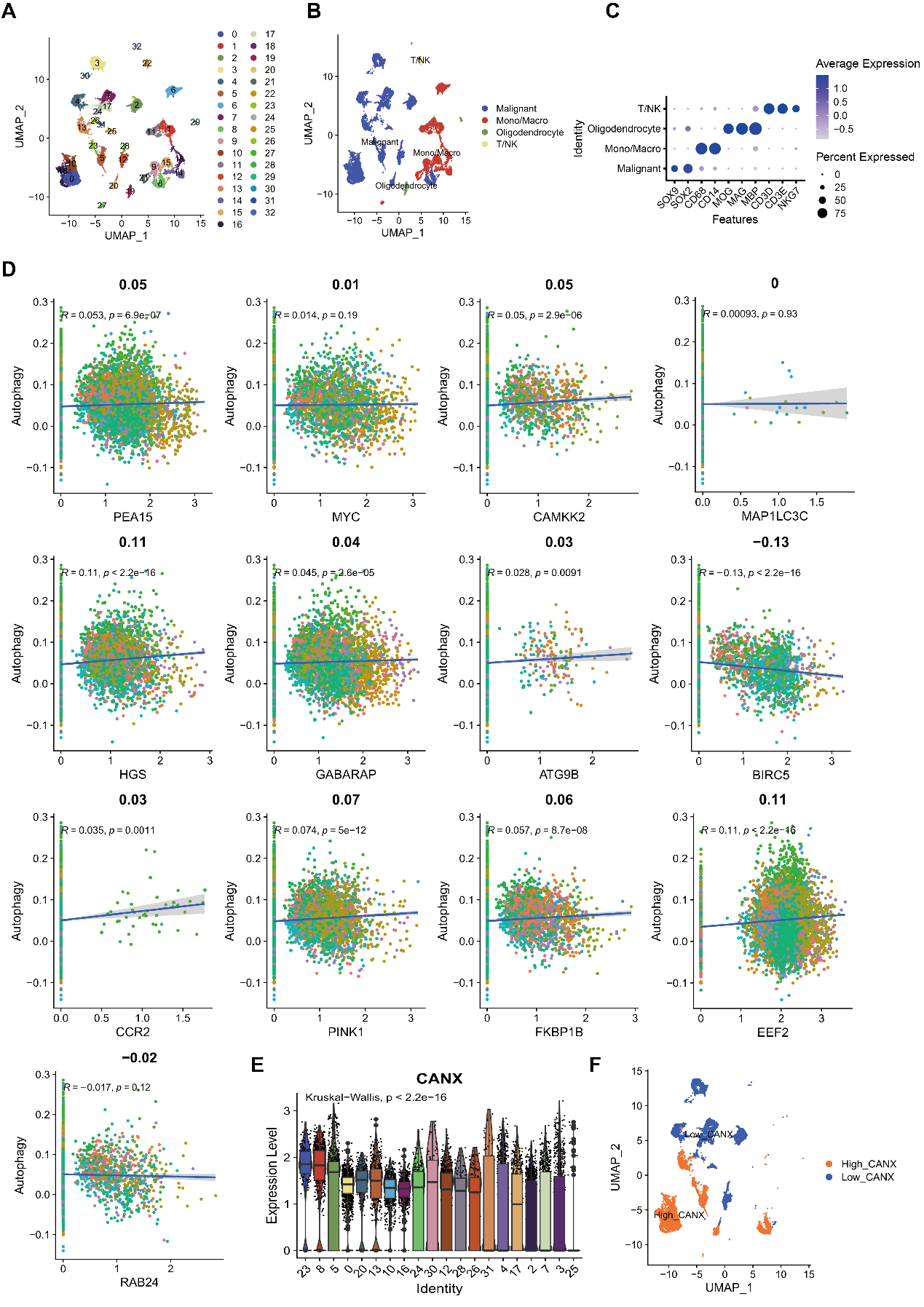


**Figure S1:** (A) UMAP plot showing the distribution of cells within glioma tissue. (B) Annotation of each cluster. (C) Utilized markers and their expression levels in each cell type. (D) Correlations between DE-ARGs significantly associated with prognosis and Autophagy scores in malignant cells. (E) RNA expression levels of CANX in different clusters of malignant cells, with “sort = TRUE”, cluster 16 and the cluster to its left categorized as High-CANX malignant cells, while the remaining clusters are classified as Low-CANX malignant cells. (F) Distribution of High- and Low-CANX malignant cells in UMAP plot.


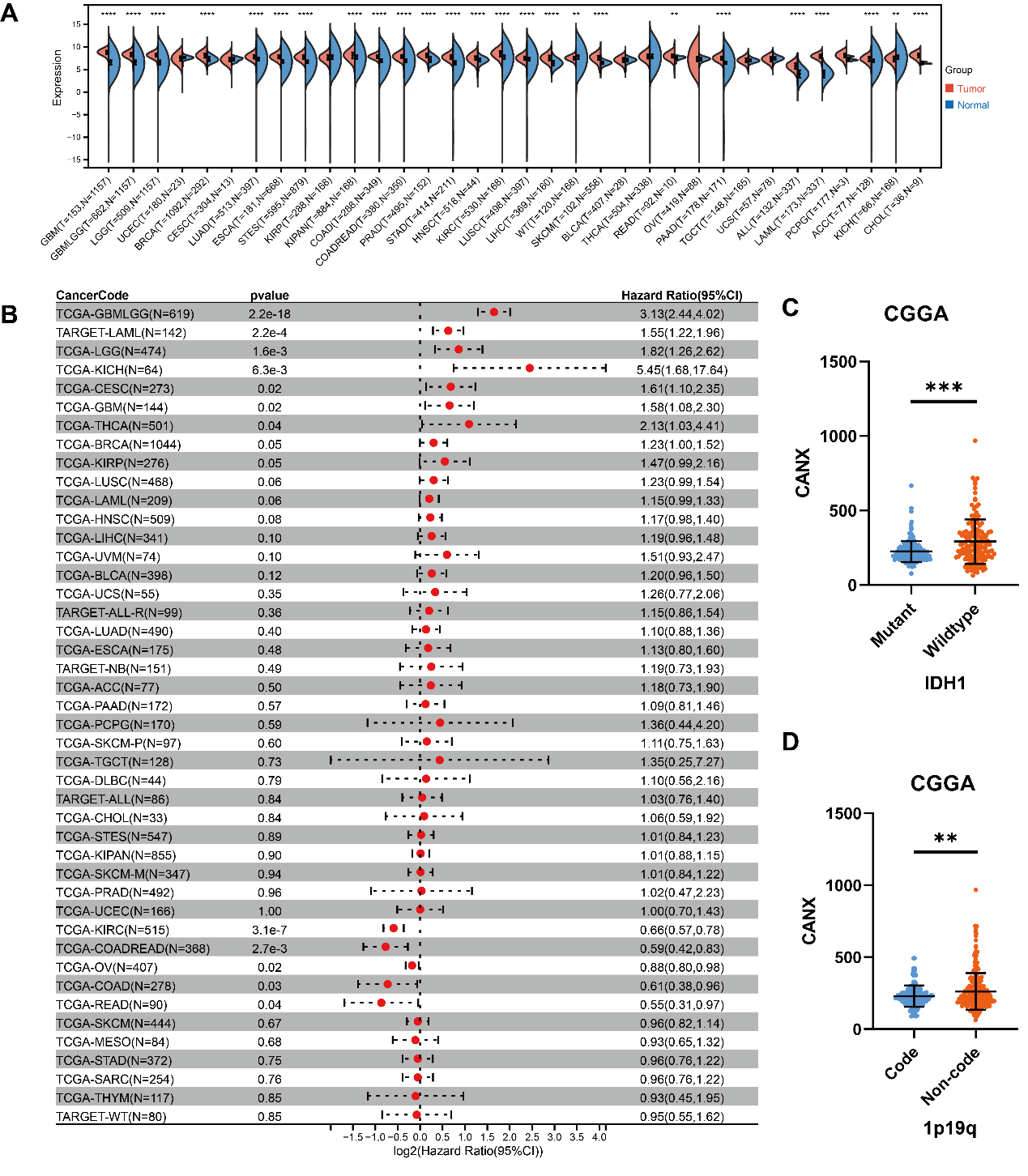


**Figure S2:** (A) Expression analysis of CANX in tumor and normal tissues via pan-cancer analysis. (B) Survival analysis according to the expression of CANX via pan-cancer analysis. (C) IDH1 mutation status corresponding to CANX expression. (D) 1p19q codeletion status in GBM patients with different CANX levels. *P < 0.05; **P < 0.01, ***P < 0.001 between the two indicated group.


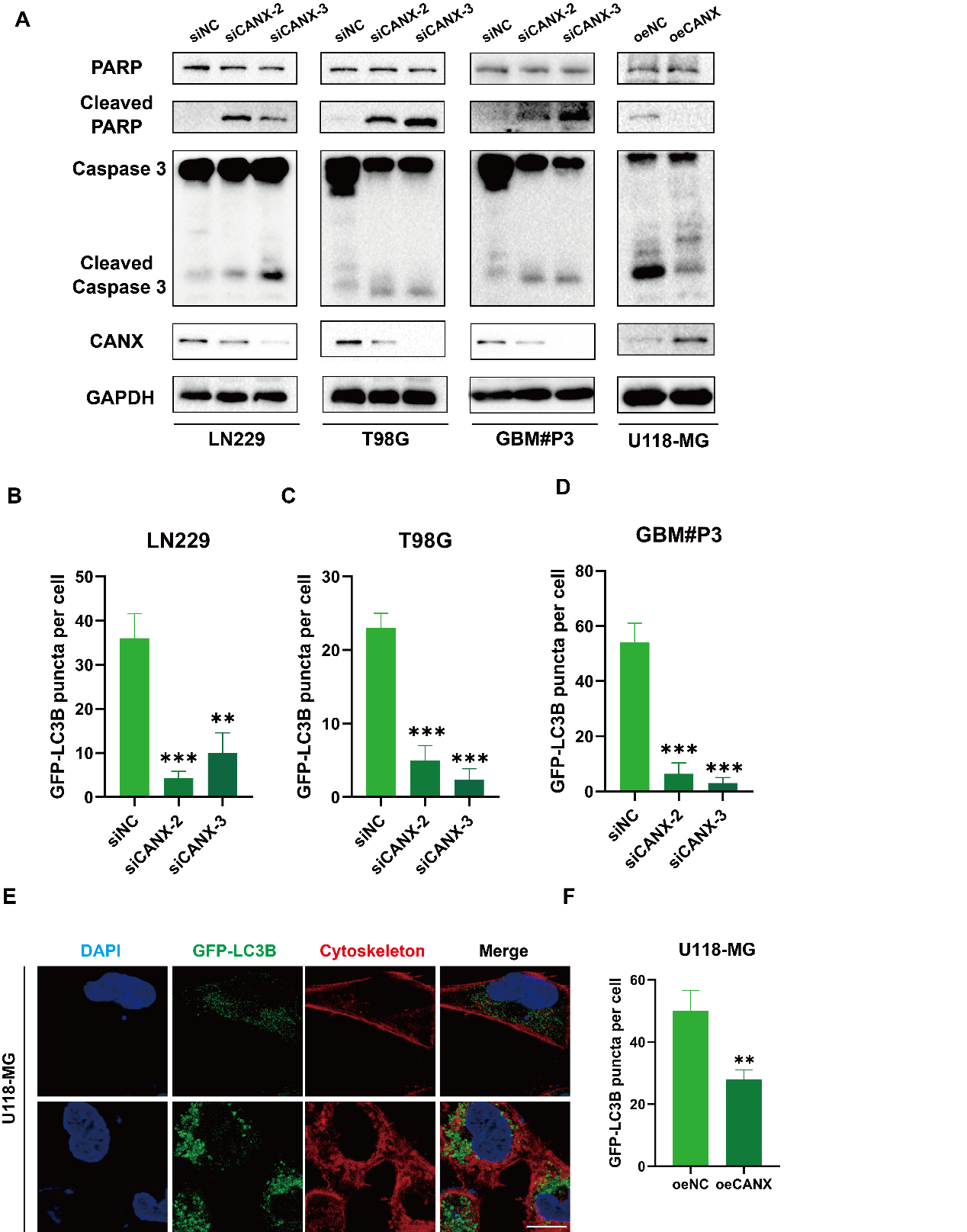


**Figure S3**: (A) Western blot analysis showing the protein level of PARP/Cleaved PARP, Caspase 3/Cleaved Caspase 3 after the knockdown of CANX. (B-D) Statistical analysis of the GFP-LC3B puncta per cell in T98G, LN229 and GBM#P3 cells treated with siNC, siCANX-2 and siCANX-3. (E) Immunofluorescence staining revealing alterations in LC3B expression following CANX overexpression in U118-MG cells via confocal microscopy (scale bar: 50 μm). (F) Statistical analysis of the GFP-LC3B puncta per cell in oeNC and oeCANX U118-MG cells. The data are shown as the means ± SDs and are representative of three independent experiments. *P < 0.05; **P < 0.01, ***P < 0.001 between the two indicated treatments.


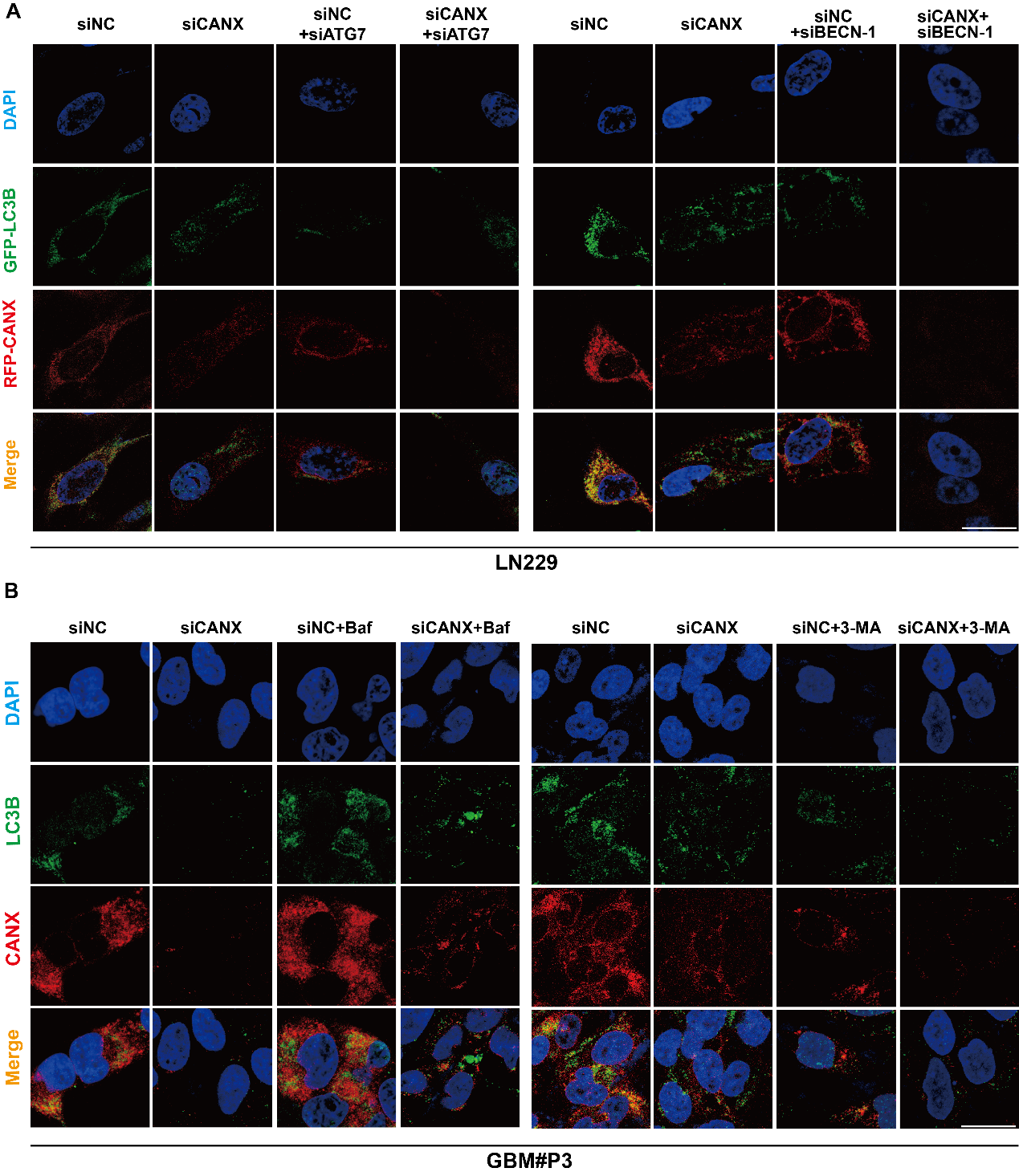
**Figure S4**: (A) Confocal microscopy analysis of immunofluorescence staining in the LN229 cell line was used to assess the impact of CANX knockdown on autophagic flux following the knockdown of ATG7 and BECN1 (scale bar: 25 μm). (B) Immunofluorescence analysis of the GBM#P3 cell line revealing the effect of CANX knockdown on autophagosome formation after treatment with Baf and 3-MA (scale bar: 25 μm).


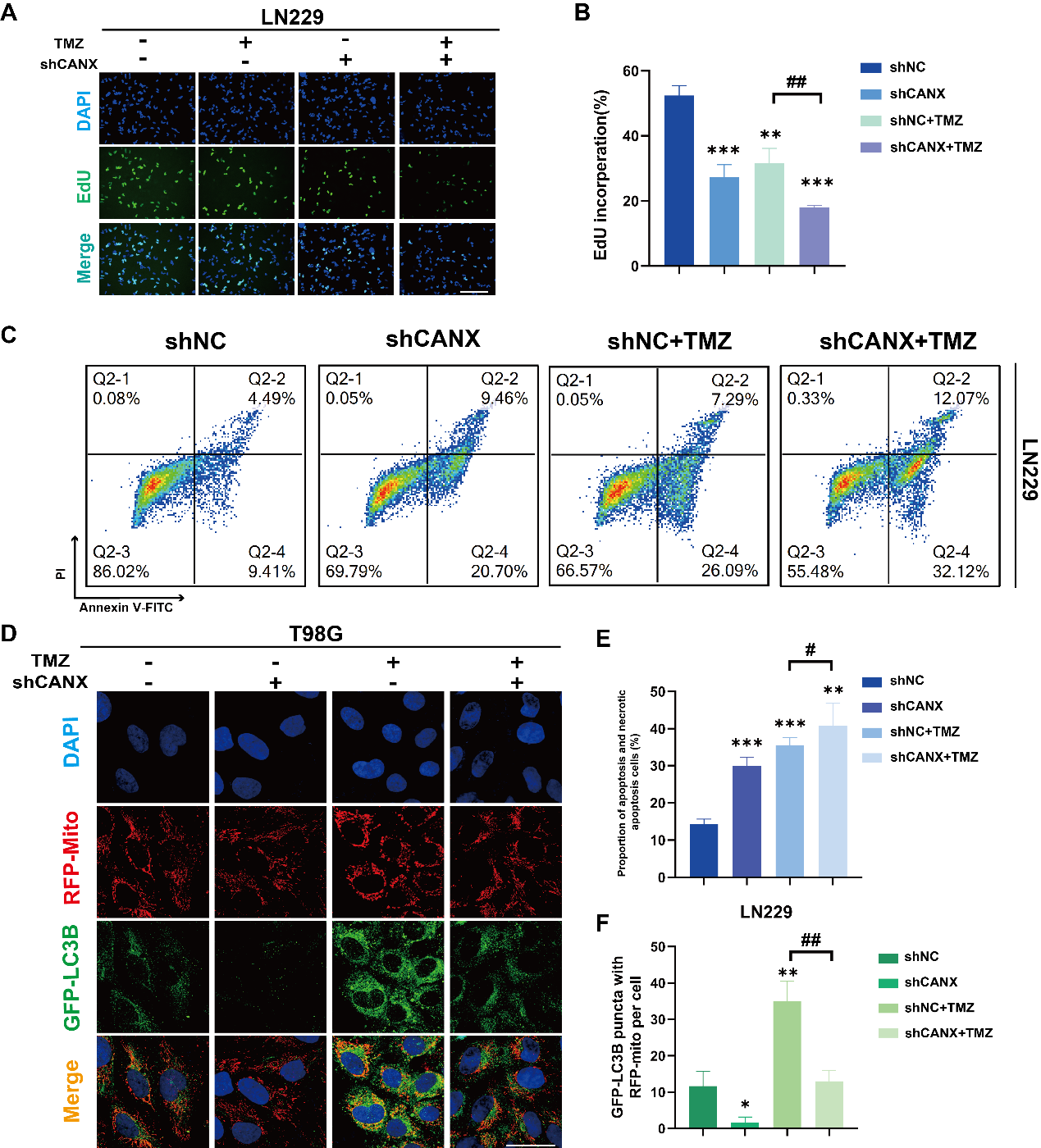


**Figure S5:** (A, B) EdU assay showing the effect of reduced CANX expression on TMZ efficacy in LN229 cells with statistical analysis (scale bar: 100 μm). (C, E) Flow cytometry analysis and statistical analysis of Annexin V-FITC and PI staining in shNC/shCANX LN229 cells with treatment of TMZ for the analysis of apoptosis. (D, F) Confocal images and statistical analysis showing changes in GFP-labeled LC3 levels in T98G cells stained with MitoTracker Red after TMZ treatment and CANX knockdown (scale bar, 40 µm). The data are shown as the means ± SDs and are representative of three independent experiments. *P < 0.05; **P < 0.01, ***P < 0.001 between the two indicated treatments.


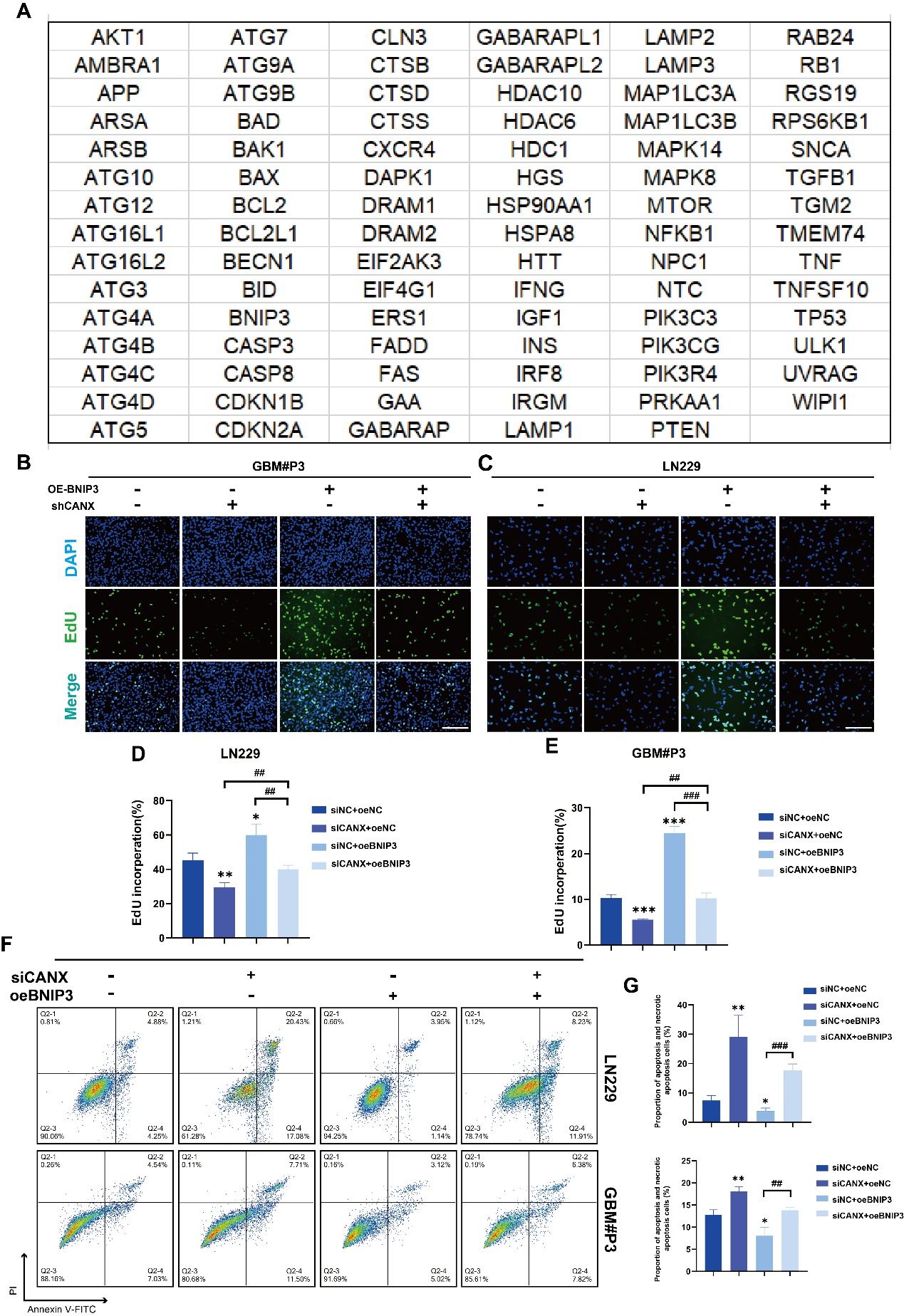


**Figure S6:** (A) Autophagy-related genes identified via a PCR array**.** (B, C) EdU assays demonstrating the effect of BNIP3 overexpression on the proliferation of GBM#P3 and LN229 cells (scale bar: 100 μm). (D, E) Statistical analysis of EdU-positive cells. (F, G) Flow cytometry analysis and statistical analysis of Annexin V-FITC and PI staining in shNC/shCANX GBM#P3 and LN229 cells overexpressing BNIP3 for the analysis of apoptosis. The data are shown as the means ± SDs and are representative of three independent experiments. *P < 0.05; **P < 0.01, ***P < 0.001 between the two indicated treatments.


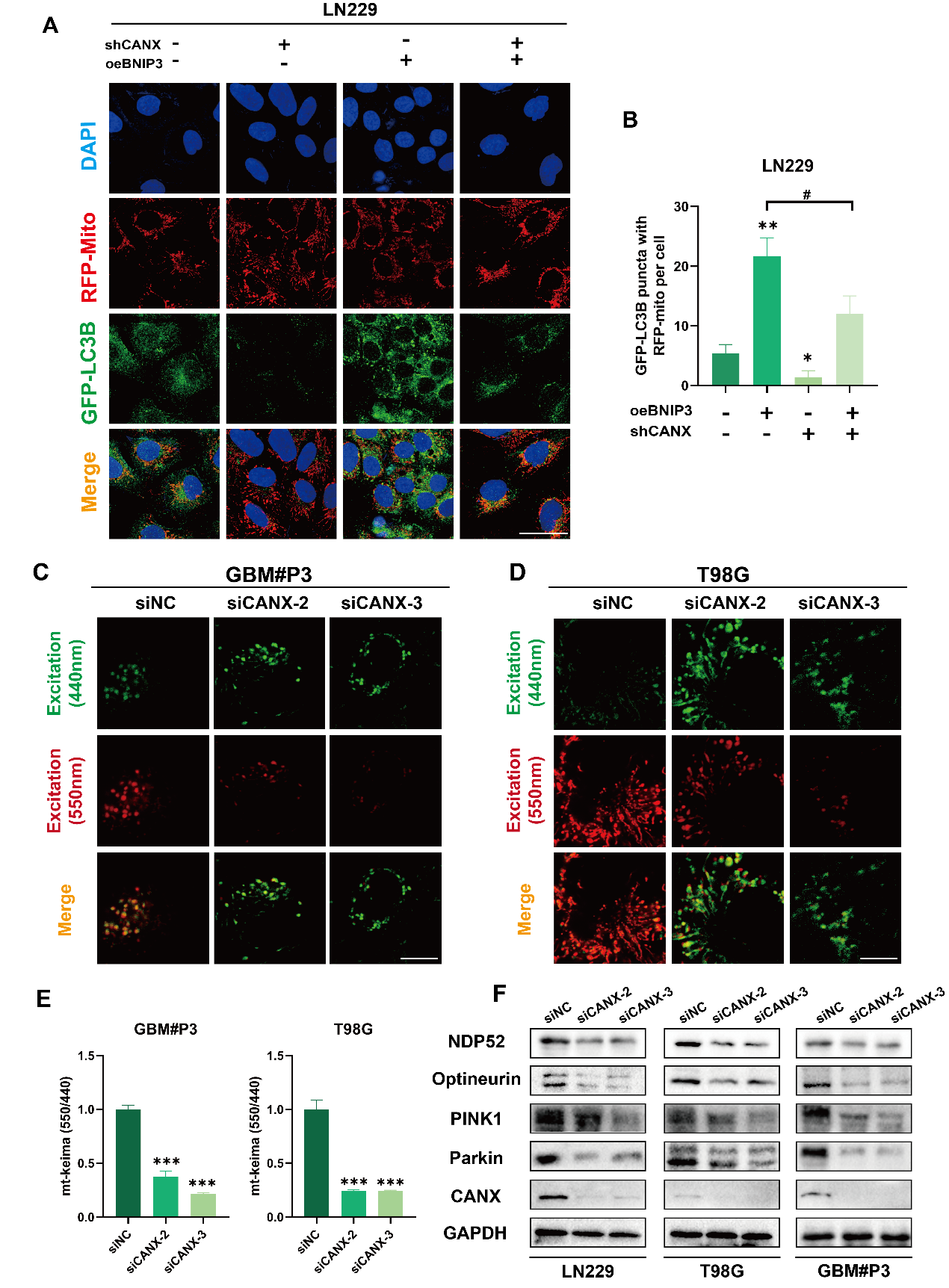


**Figure S7:** (A) Mitochondrial probes were used to assess changes in mitophagy following CANX knockdown and BNIP3 overexpression in LN229 cells (scale bar: 40 μm). (B) Statistical analysis of the colocalization of GFP-LC3B puncta with RFP-mito in each cell. (C, D) Representative confocal images are of GBM#P3 and T98G cells expressing mt-Keima transfected with siNC or siCANX (scale bar: 10 μm). (E) Statistical analysis of the ratio of fluorescence intensity at 550nm excitation versus 440nm excitation. (F) Western blot analysis showing the protein level of NDP52, PINK1, Optineurin and Parkin after the knockdown of CANX. The data are shown as the means ± SDs and are representative of three independent experiments. *P < 0.05; **P < 0.01, ***P < 0.001 between the two indicated treatments.


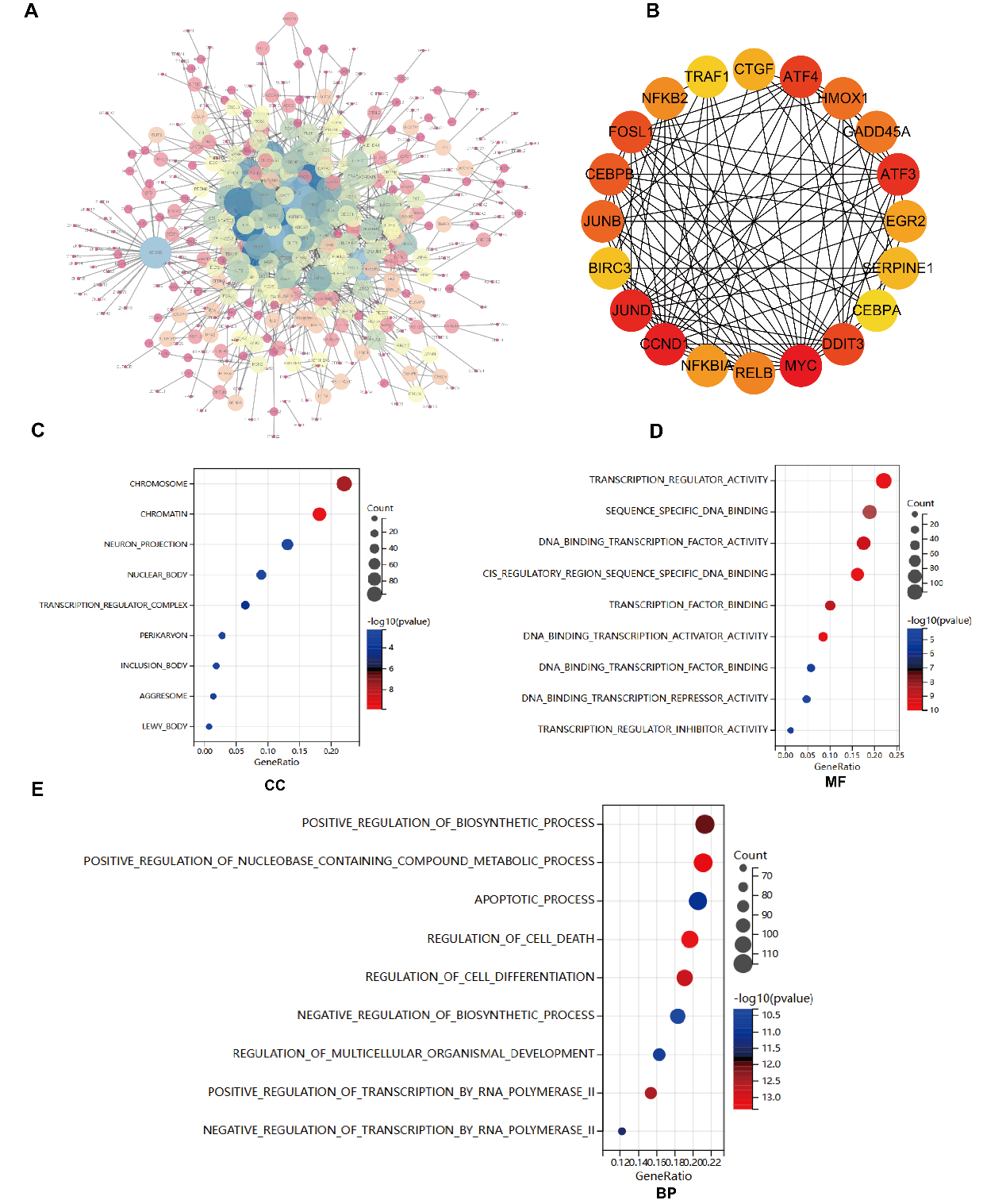


**Figure S8:** (A) PPI network of CANX-related DEGs. (B) The top 20 CANX-related hub genes. (C-E) GO enrichment analysis (CC, MF and BP terms) of the DEGs related to CANX.


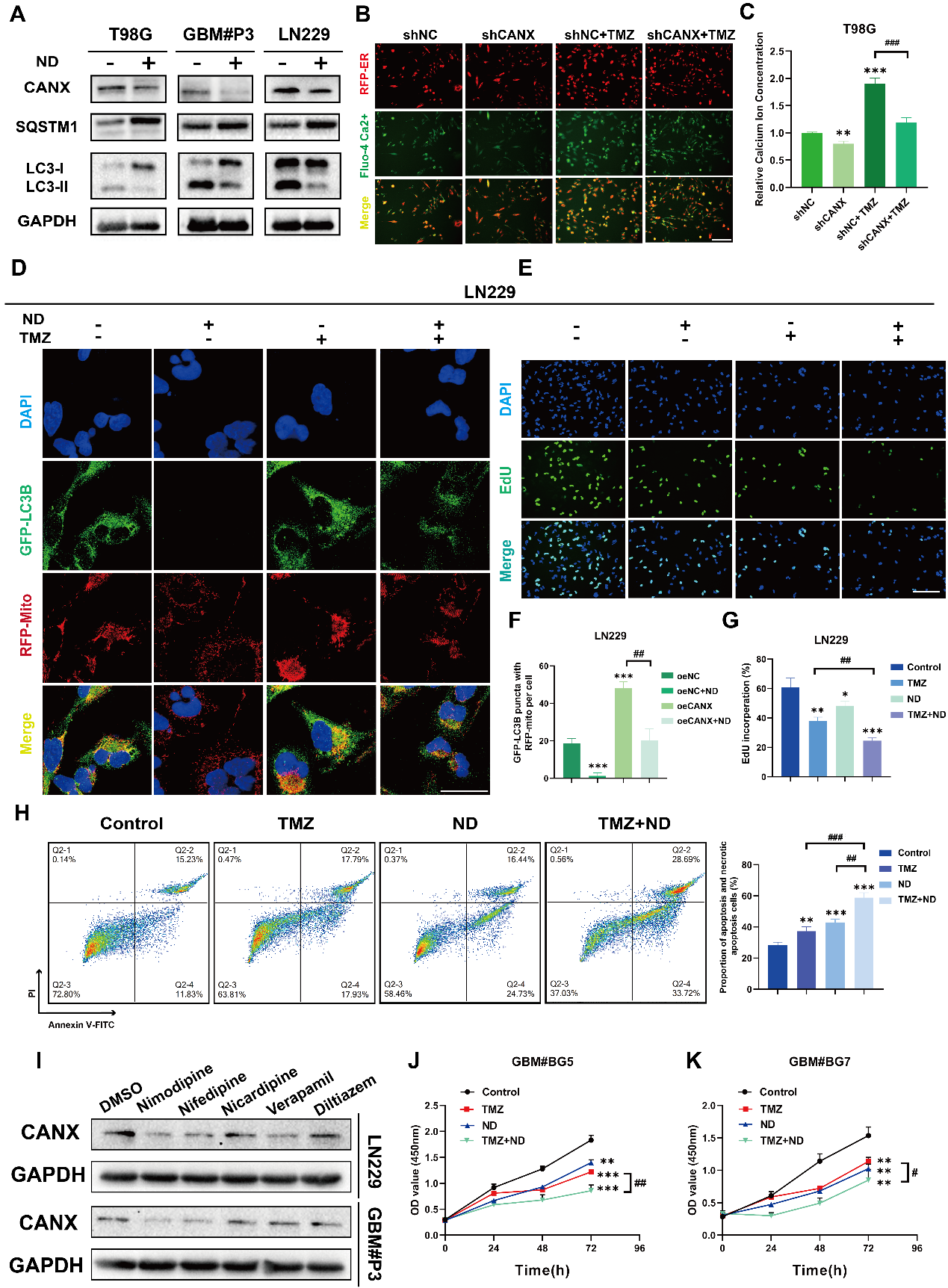


**Figure S9**: (A) Western blot analysis showing changes in CANX, SQSTM1 and LC3B expression in GBM#P3, T98G, and LN229 cells after treatment with ND. (B) Analysis of the Ca^2+^ fluorescence intensity in GBM#P3 cells (shNC/shCANX group) treated with TMZ (scale bar: 80 μm). (C) Quantitative analysis of the Ca²^+^ fluorescence intensity in T98G cells (shNC/shCANX) before and after TMZ treatment via a microplate reader. (D, F) Representative confocal images showing changes in GFP-labeled LC3 levels in LN229 cells stained with MitoTracker Red after treatment with ND and TMZ (scale bar: 40 μm). (E, G) EdU assay showing the proliferation of GBM#P3 and T98G cells after treatment with TMZ and ND (scale bar: 40 μm). (H) Flow cytometry analysis of apoptosis levels in LN229 cells after treatment with TMZ or ND. (I) The Western Blot results showed the expression of CANX in LN229 and GBM#P3 cells after the application of different types of calcium channel blockers. (J, K) The CCK-8 experiment revealed the activity of GBM#BG5 and GBM#BG7 cells when using ND and TMZ. The data are shown as the means ± SDs and are representative of three independent experiments. *P < 0.05; **P < 0.01, ***P < 0.001 between the two indicated treatments.


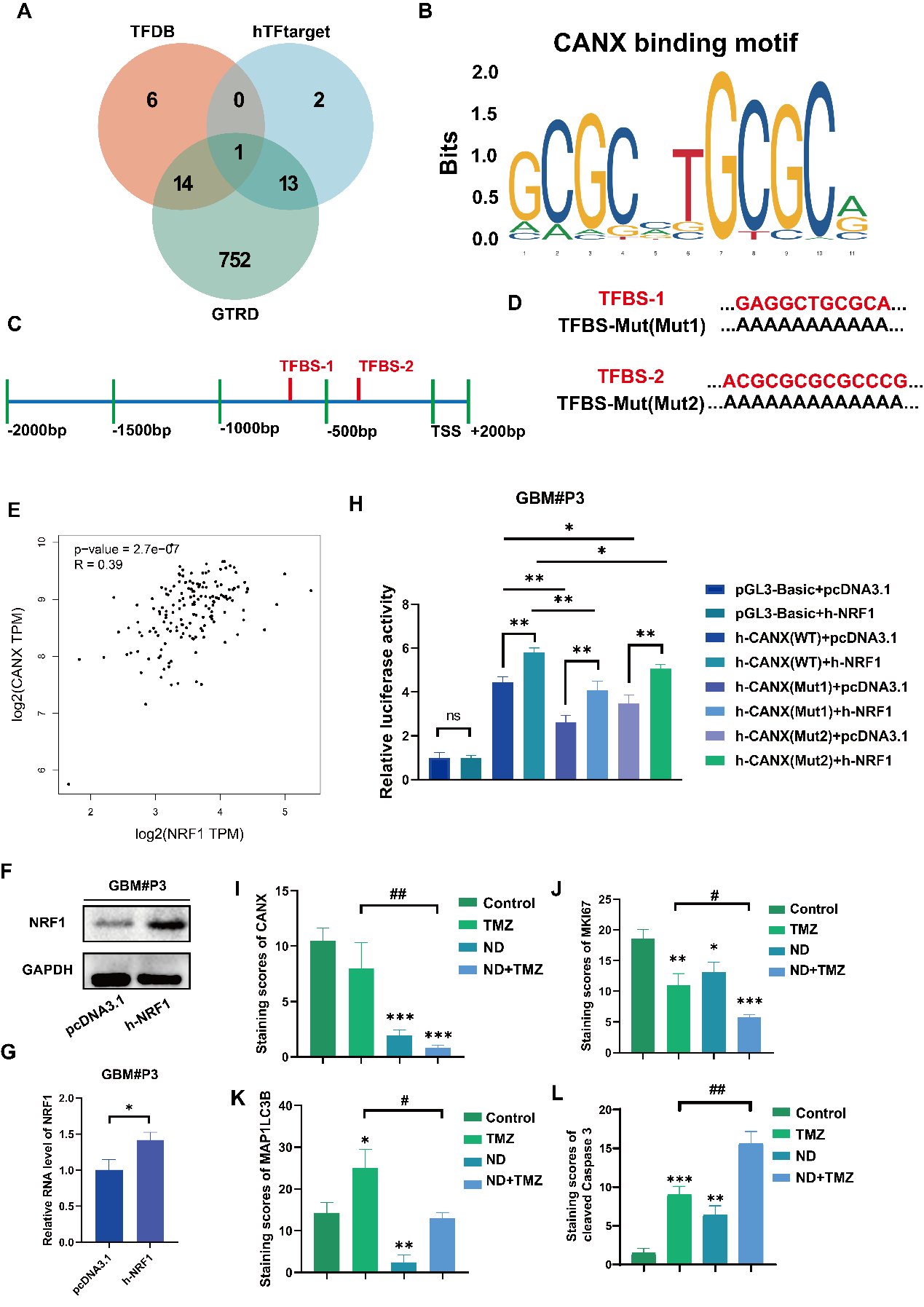


**Figure S10:** (A) A Venn diagram illustrates the transcription factors predicted to interact with the CANX promoter by hTFtarget, TFDB, and GTRD databases. (B) The CANX-binding motif of NRF1. (C) The predicted potential binding site of NRF1 on the CANX promoter. (D) Potential binding sites of NRF1 on the CANX promoter sequence and the corresponding mutations. (E) Correlation analysis of NRF1 and CANX expression levels in glioma patients. (F, G) Western blot and qPCR showed the relative protein and RNA expression level of NRF1 with pcDNA3.1 and h-NRF1 treatment. (H) CANX promoter (WT, Mut1 and Mut2) luciferase activity was detected by dual-luciferase assays. (I- L) Statistical analysis of MKi67, LC3B, CANX, and cleaved Caspase 3 expression in tumors from each group via immunohistochemical staining. The data are shown as the means ± SDs and are representative of three independent experiments. *P < 0.05; **P < 0.01, ***P < 0.001 between the two indicated treatments.
